# Project YES! Youth Engaging for Success: A randomized controlled trial assessing the impact of a clinic-based peer mentoring program on viral suppression, adherence and internalized stigma among HIV-positive youth (15-24 years) in Ndola, Zambia

**DOI:** 10.1371/journal.pone.0230703

**Published:** 2020-04-02

**Authors:** Julie A. Denison, Virginia M. Burke, Sam Miti, Bareng A. S. Nonyane, Christiana Frimpong, Katherine G. Merrill, Elizabeth A. Abrams, Jonathan K. Mwansa

**Affiliations:** 1 Department of International Health, Johns Hopkins Bloomberg School of Public Health, Baltimore, Maryland, United States of America; 2 Arthur Davison Children’s Hospital, Ndola, Zambia; International AIDS Vaccine Initiative, UNITED STATES

## Abstract

**Background:**

Youth-led strategies remain untested in clinic-based programs to improve viral suppression (VS) and reduce stigma among HIV-positive adolescents and young adults (AYA) in sub-Saharan Africa. In response, Project YES! placed paid HIV-positive youth peer mentors (YPM) in four HIV clinics in Ndola, Zambia including a Children’s Hospital (pediatric setting), an adult Hospital and two primary care facilities (adult settings).

**Methods:**

A randomized controlled trial was conducted from December 2017 to February 2019. Consecutively recruited 15 to 24-year-olds were randomly assigned to an intervention arm with monthly YPM one-on-one and group sessions and optional caregiver support groups, or a usual care comparison arm. Survey data and blood samples were collected at baseline and at the six-month midline. Generalized estimating equation models evaluated the effect of study arm over time on VS, antiretroviral treatment (ART) adherence gap, and internalized stigma.

**Results:**

Out of 276 randomized youth, 273 were included in the analysis (Intervention n = 137, Comparison n = 136). VS significantly improved in both arms (I:63.5% to 73.0%; C:63.7% to 71.3.0%) [OR:1.49, 95% CI:1.08, 2.07]. In a stratified analysis intervention (I:37.5% to 70.5%) versus the comparison (C:60.3% to 59.4%) participants from the pediatric clinic experienced a relative increase in the odds of VS by a factor of 4.7 [interaction term OR:4.66, 95% CI:1.84, 11.78]. There was no evidence of a study arm difference in VS among AYA in adult clinics, or in ART adherence gaps across clinics. Internalized stigma significantly reduced by a factor of 0.39 [interaction term OR:0.39, 95% CI:0.21,0.73] in the intervention (50.4% to 25.4%) relative to the comparison arm (45.2% to 39.7%)

**Conclusions:**

Project YES! engaged AYA, improving VS in the pediatric clinic and internalized stigma in the pediatric and adult clinics. Further research is needed to understand the intersection of VS and internalized stigma among AYA attending adult HIV clinics.

**Trial registration:**

ClinicalTrials.gov NCT04115813.

## Introduction

Adolescents and young adults (AYA) living with HIV access HIV care less and have lower rates of adherence to antiretroviral therapy (ART) and viral suppression (VS) compared to adults [1–7]. While there has been a sustained international call to provide support and tools for HIV self-management behaviors among adolescents as they transition into adulthood [5, 8, 9], including adhering to ART, practicing safer-sex behaviors, and transitioning to adult care [10–14], AYA in sub-Saharan Africa (SSA) do not routinely have access to youth-specific services or opportunities to build life skills [5]. To address the unique challenges HIV-positive AYA face during this distinct developmental stage characterized by physical, social, emotional, and cognitive development [15] and to maximize resilience among youth, PEPFAR/USAID guidance on the transition of care for adolescents living with HIV [16] emphasizes several key issues:

- For some adolescents living with HIV, the transition of care process includes a physical transition from a pediatric or adolescent model of care to an adult facility;
- Many adolescents living with HIV in SSA already receive care in adult HIV clinics, yet still need support to develop the skills to self-manage their HIV; and
- The process of transition of care and HIV self-management is complex and a “multifaceted, active process” that must “attend to the medical, psychosocial, and educational or vocational needs of adolescents” [17].

Despite our knowledge of the needs of transitioning adolescents drawn from other chronic illnesses, as well as the impact families and peers have on AYA health [18–20], there is little published literature on interventions to support HIV-positive youth and their families in SSA [21]. For example, a 2015 review found only 14 studies on HIV-positive adolescents transitioning to adult care, all of which were conducted in the US or UK and the majority of which were qualitative studies with sample sizes of 50 participants or fewer [22]. A 2016 systematic review examined the literature to assess the effectiveness of self-management interventions for young people across chronic illnesses. Out of 42 randomized controlled trials included in the review, none were conducted in SSA. The authors note that most interventions focused on the medical aspects of self-management rather than psycho-social issues [23].

To address this gap in evidence-based interventions, this study tested a peer mentoring program that drew upon the “Five C’s” of positive youth development: competence, confidence, connection, character and caring. Particularly salient principles are confidence, the internal sense of overall positive self-worth, and connection, the positive bonds youth have with peers, family, community and institutions [24–26]. The study also drew upon constructs from Social Cognitive Theory including self-efficacy to perform a behavior, agency to change one’s situation and observational learning with peer mentors acting as role models [27, 28].

Peer mentors, especially those who are well trained and given paid positions, have been effective in other settings, such as the mothers2mothers program in South Africa which places mentor mothers in clinics to work with HIV-positive pregnant women to prevent mother-to-child HIV transmission [29] or the Restless Development program in Zambia that placed young adults in schools to mentor students on life skills [30]. In addition, our prior research among 311 HIV-positive adolescents in Ndola, Zambia found that 88% of participants were interested in having a mentor, such as a young adult living with HIV, with whom to talk [31]. That same study found that 93% of interviewed youth wanted to attend group sessions with their HIV-positive peers. Peer group interventions have shown some promise in reducing HIV-related stigma in SSA among HIV-positive adults [32] and supporting ART adherence among adolescents [33, 34].

Drawing on this existing evidence, Project YES! trained and hired youth peer mentors living with HIV as paid staff and placed them in four HIV clinics. By having mentors work with youth individually and in group sessions over time, this research embodies the approach that healthcare transition is “a continuum and not separate, discrete moves from pediatric to adolescent to adult clinic settings” [35]. We hypothesize that such youth-led strategies are needed to effectively support AYA to successfully adhere to ART and decrease internalized stigma in order to achieve viral suppression and the 2030 UNAIDS 95-95-95 goals [36].

## Materials and methods

### Study design and setting

A randomized controlled trial was conducted among AYA living with HIV attending four HIV clinics in Ndola, Zambia–one children’s hospital, one adult hospital, and two primary health care facilities. The two hospitals each had HIV clinics with adolescent focused days and hours. The two primary care clinics offered HIV services on specific days only. We estimated that with a sample size calculation of 144 per group, we had 85% power to detect a difference of 20% (an increase from 50% to 70%) between the proportions VS in the intervention versus the comparison arms at midline, with Type 1 error of 5% and 20% loss to follow-up.

The trial was registered retrospectively at clinicaltrials.gov (NCT04115813), once authors became aware of this requirement for publication. The authors confirm that all ongoing and related trials for this intervention are registered; there are no ongoing trials related to this study.

### Study participants

Study participants were drawn from a population of AYA ages 15–24 attending the four HIV clinics described above. AYA also had the option of inviting adult caregivers to the program as well.

#### Eligibility

Eligible youth were between the ages of 15 to 24 years, aware of their HIV status, had been on ART for six or more months, spoke Bemba or English, and planned to be available to attend study activities over the next 18 months as needed. Exclusion criteria for youth included being too sick to participate, attending boarding school, having a sibling already enrolled in the study (one youth per household), or having participated in a prior adolescent/caregiver intervention study held at two of the study sites [31].

#### Sampling and recruitment

A systematic sampling approach based on clinic attendance data from Zambia’s electronic health record system (SmartCare) was initially used by trained study staff to recruit every other eligible youth, ages 15 to 24, in the children’s hospital and 15 to 19 in the adult clinic settings, while they attended clinic. Due to slow recruitment and the receipt of revised, decreased SmartCare estimates, the study team amended the protocol to recruit every eligible youth who attended the clinic as a consecutive sample and increased the age of participation at all clinics to age 24. If determined to be eligible and interested, trained interviewers would facilitate the informed consent process, enroll the AYA participant and collect baseline data.

### Randomization

After completing the baseline survey, all participants were randomized to either the intervention or the comparison arms. A stratified randomly permuted block randomization (block sizes 4 and 6) was used to generate the randomization scheme. Randomization was stratified by sex and age within each of the four clinics for a total of 16 strata. A biostatistician unaffiliated with the study generated a random allocation list separately for each stratum using `ralloc’ command [37] in STATA statistical software [38]. Pre-labeled, opaque, sealed envelopes randomly sequenced were opened by trained interviewers in the presence of the participants in numeric order to assign treatment group in a 1:1 allocation ratio. This process resulted in 139 participants assigned to the intervention and 137 assigned to the comparison study arm for analyses.

### Laboratory testing procedures

At baseline, participants also underwent HIV-1 RNA viral load testing using the CobasAmpli-prep/CobasTaqman 96 machine (Roche Systems, Germany). Blood samples with viral loads of 1,000 copies or more per milliliter (virologic treatment failure) were further subjected to an HIV drug resistance test using an Applied Biosystems Genetic Analyzer model3500XL (Hitachi, Japan) [testing protocols available on dx.doi.org/10.17504/protocols.io.bcc7iszn]. Efforts were made to contact and switch drugs for all participants who indicated resistance to a drug in their current ART regimen before the start of the intervention, regardless of study arm assignment.

### Data collection procedures

Recruitment, enrollment and baseline data collection occurred from December 2017 to May 2018. Data, including ART start-date and pre-ART CD4 cell count, were also collected from participants’ medical charts. A 6-month midline follow-up assessment consisting of a survey and blood draw was conducted from October 2018 to February 2019. The analyses presented in this paper are based on the 6-month midline data. After the 6-month midline the study proceeded with a cross-over design with all participants receiving a form of the intervention, the results of which will be presented in a separate manuscript.

#### Project YES! intervention arm

The Project YES! intervention arm consisted of several components. First, all participants received an orientation meeting with a health care provider (HCP), their assigned youth peer mentor (YPM), and an adult caregiver (if invited by the youth participant). During the orientation meeting, the HCP introduced the study participant to their YPM, reviewed the viral load test result, and shared the Project YES! goal of supporting the youth to maintain or achieve a suppressed viral load. In the second half of the orientation meeting, the YPM and youth participant met separately while the HCP met with the caregiver, if present, to discuss any questions related to Project YES! or the youth’s HIV care. Following the orientation meeting, intervention arm participants continued to meet with their assigned YPM for one-on-one meetings once per month over six months. Participants were also invited to monthly youth group meetings, the first of which was required in order to encourage those youth to try a group format. These monthly youth group meetings were facilitated by YPM, with a HCP invited to attend when clinical or technical information was needed.

Simultaneously, adult caregivers of youth participants were offered a total of three caregiver group meetings, held every other month. These caregiver group meetings were designed to provide adults with enhanced knowledge and skills to support their youth living with HIV.

The project also provided Youth and Caregiver Journals that participants could use to track adherence, engage with educational topics, and reflect on their journeys. These journals drew upon the USAID-funded AIDSTAR Transition Toolkit and the Positive Connections Youth Group Manual [16, 39]. After the midline data collection, as described above, intervention arm participants started a maintenance phase.

Youth intervention participants attending the children’s hospital were additionally assessed for a physical transition to the adult hospital before the start of the intervention. This assessment was based on clinical eligibility (viral load status and opportunistic infections) and psycho-social factors (i.e. does not have a challenge they are facing, such as moving homes or a recent death in the family). Participants who were eligible to be transferred were then invited to attend a group transition meeting prior to the start of the intervention to tour the adult clinic with their HIV-positive peers and familiarize themselves with the new clinic and assigned YPMs. The study team also planned to have a pediatric clinic staff member attend the first two clinical visits of the newly transitioned youths to ease the transition process. However, this program component did not occur systematically for every transitioned youth.

#### Youth peer mentoring training

YPMs were HIV-positive young adults between the ages of 21–26 years who had successfully transitioned to self-management. These peer mentors underwent a capacity-building process. First, they participated in an intensive two-week pre-service training lead by a Training and Capacity Building Specialist that prepared them to be skilled, valued, and paid youth mentors and employees of the health care system. The pre-service training also included opportunities for the YPM to reflect on their own experiences of living with HIV and assess their own self-management and self-care practices. Second, the YPMs had a month of practice meetings with HIV-positive youth 18 years and older prior to the start of the intervention. Third, about midway through Project YES!, the YPM underwent an in-service training with the same Training and Capacity Building Specialist to reinforce and expand their skills. Fourth, the YPMs met weekly as a group to discuss challenges, approaches, and ideas. Fifth, throughout the study, YPMs were able to rely on active supportive supervision from study team members.

#### Comparison arm

Participants in the comparison arm received the standard of care for adolescents and young adults as offered at the HIV clinics, including regular clinic visits and the option of joining monthly youth group meetings. After the midline data collection, comparison arm participants started the Project YES! intervention as described above, including transitioning eligible youth from the children’s hospital to the HIV clinic in the adult hospital.

### Measures

The pre-specified outcomes included viral suppression, ART adherence and internalized stigma.

**Viral suppression** was defined as a viral load test result of <1,000 copies/mL versus a viral load test result of ≥1,000 copies/mL.

#### ART adherence

An ART adherence treatment gap was assessed at the two time points through two questions: “*In the past three months*, *did you have a day when you did not take any ART drugs*?” and “*What were the most days in a row that you missed swallowing your drugs in the past three months*?” [40, 41]. A binary outcome was generated so that a participant was considered to *not* have a treatment gap if they said no to missing any full days of ART drugs in the past three months, or if they said yes and only missed one day. Otherwise, they were considered to have an ART adherence treatment gap defined as 48 consecutive hours or more in the past three months. If the participant was missing answers to both these questions, their ART adherence treatment gap outcome was considered missing.

#### Internalized stigma

Internalized stigma was measured at both time points using three agree/disagree questions from the Internalized AIDS Stigma Scale (IA-RSS) [42], that have been used in a previous study among a population of HIV-positive adolescents in Ndola, Zambia [41]. This measure asks participants to either “agree” or “disagree” with each of the following three statements: (1) *You feel guilty that you are HIV positive;* (2) *You are ashamed that you are HIV positive; and* (3) *You sometimes feel worthless because you are HIV positive*. From this data, a binary outcome was generated with “1” indicating that the participant answered “agree” to at least two of the three questions, and “0” otherwise [41]. If answers to all three were missing, this outcome was considered missing.

Independent variables adjusted for in the analyses included sex (male vs. female), age (categorized as 15–19 versus 20–24) and enrollment site (the clinic where the AYA was recruited and enrolled). In addition, we also assessed intervention exposure as defined by the number of one-on-one peer mentoring sessions and the number of group sessions each youth attended, as well as if the youth had a caregiver attend any of the Project YES! caregiver sessions. Finally we assessed potential contamination by asking the following questions at the 6-month midline assessment of the intervention arm participants: “Have you shared anything you learned or talked to your YPM about with other patients at the clinic who are part of Project YES!, but who are in the other group who will start meeting the peer mentor second”. We also asked the comparison arm participants “has anyone from the group who has been meeting with a youth peer mentor first shared anything they learned or talked about with a peer mentor with you?” Participants who responded yes to these questions were then read a list of Project YES! intervention topics to see if program content was discussed.

### Statistical analysis

This paper presents the primary analysis comparing the intervention versus comparison arms using baseline and 6-month midline data We summarized baseline socio-demographic and clinical characteristics of the study sample using counts and percentages for categorical variables and means and standard deviations for continuous variables. The pre-determined outcomes for the primary analysis comparing the interventions versus the comparison arms over time (baseline versus midline) were VS (<1,000 copies/mL), ART adherence treatment gap, and internalized stigma among youth participants.

#### Viral suppression

We fitted generalized estimating equation (GEE) models with a logit link and an unstructured correlation structure to account for the correlation among individuals’ measurements. As a robustness check, we also fitted a GEE model weighted by the inverse probability of truncation, which does not assume that the losses of participants by midline were completely at random. The primary analysis model included study arm as the main exposure of interest and adjusted for time point (midline versus baseline), time point by arm interaction, and the variables used for stratification in the randomization: sex, age and enrollment site. We evaluated the potential mediation effect of the length of time between baseline and midline measurement using a generalized structural equation model, with VLS at midline as the outcome, adjusting for age, sex and enrollment site. A stratified analysis by pediatric versus adult HIV treatment sites, which had been specified a priori, was also conducted using a GEE model.

#### ART adherence

We generated proportions of participants with a gap at both time points (baseline and the 6-month midline) by study arm. We then fitted a GEE model to evaluate the effect of study arm on having an ART adherence treatment gap, adjusting for time and stratification variables.

#### Internalized stigma

We calculated a Cronbach’s alpha to evaluate internal consistency among the three internalized stigma questions. A GEE model was fitted to evaluate the effect of the intervention on internalized stigma.

#### Exposure

We conducted Chi-squared tests for the null hypotheses that attending at least five out of six group meetings or having a caregiver attend any of the meetings was associated with VS at midline.

### Ethical considerations

This study was reviewed and approved by the ERES Converge Institutional Review Board in Zambia, the Zambia Ministry of Health through the National Health Research Authority, and the Johns Hopkins Bloomberg School of Public Health Institutional Review Board in the United States. Participants were reimbursed 50 Kwacha (about 5.00 USD) for their time and travel to the clinic for study-related appointments. In addition, intervention participants were provided snacks during monthly group meetings.

In accordance with Zambian law, participants 15 to 17 years old provided written assent and their parent/guardian provided parental permission [43]. Trained interviewers administered tablet-based baseline surveys using Magpi software that included questions about various background characteristics and HIV-related health outcomes as well as experiences of violence and suicide ideation. The interviewers referred participants who reported severe violence or thoughts of suicide in the past week to HCPs for additional care.

## Results

**Fig 1:**
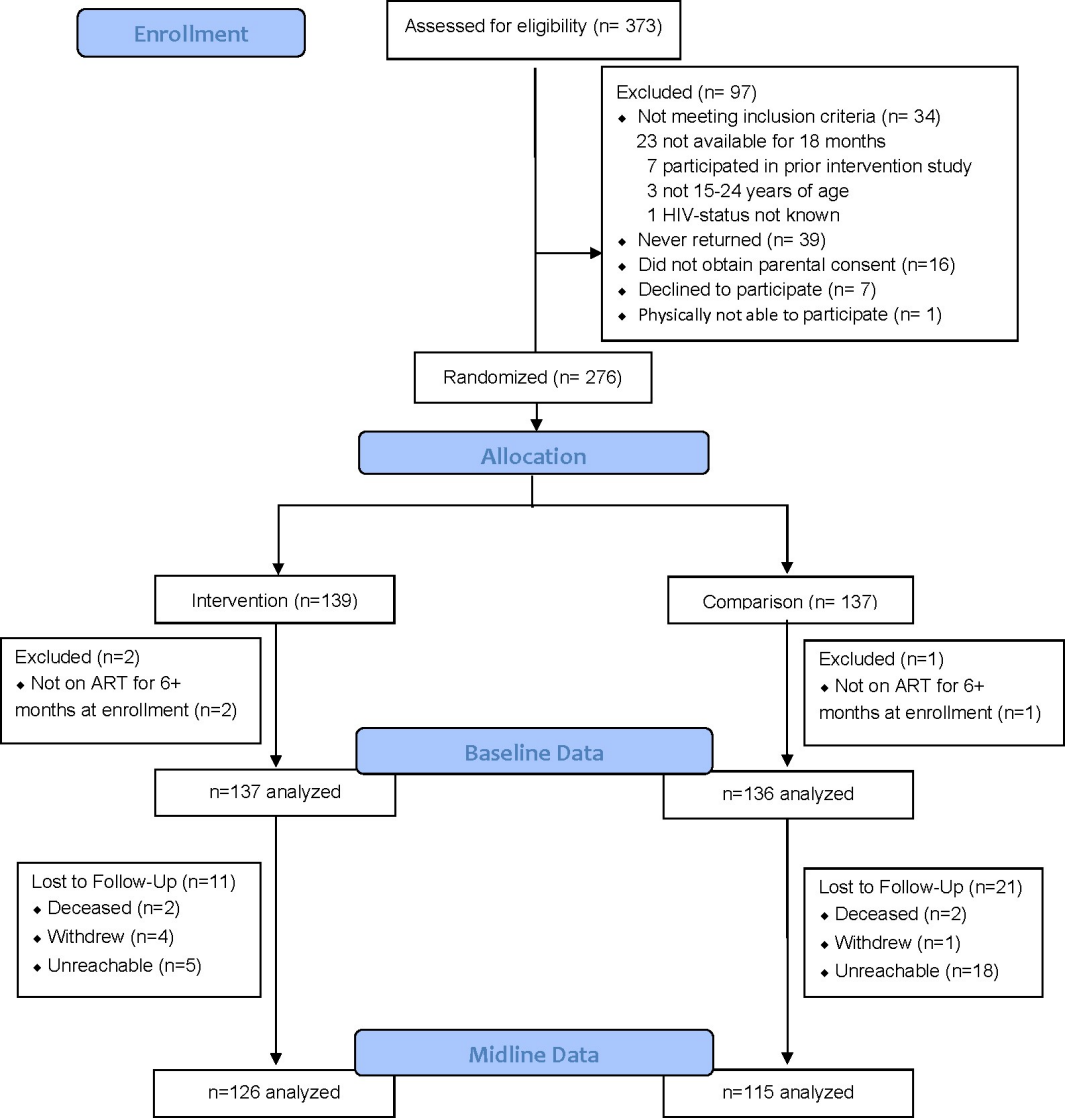
Consort diagram.

During recruitment, the research assistants approached 373 youth the HCPs confirmed were aware of their HIV status, of whom 74% (n = 276) enrolled into the study (Fig 1). Out of the 97 who did not enroll, 34 were not eligible, 39 requested to join later and then were not reachable/never returned, 16 did not obtain parental permission, and 7 declined for various reasons including not being interested or not having travel money.

During data cleaning it was determined that three enrolled participants had been on ART for less than six months. These three participants were subsequently excluded from analyses for not meeting the eligibility criteria, bringing the analysis sample to 273. At baseline, the 137 participants randomly assigned to the intervention arm and the 136 to the comparison arm were balanced with respect to key demographic and clinical characteristics (Table 1). The average age was 19 years (range 15–24) and 162 (59.3%) were female. The majority, 240 (87.9%) had completed at least primary school. Most (n = 198, 72.5%) self-reported having acquired HIV perinatally. The median CD4 count prior to ART initiation was 284 (IQR 154, 453) among the 187 participants with available pre-ART CD4 cell count data and the median number of years on ART was 7.3 (IQR 3.5, 9.9). Within each study arm, the time between baseline and midline viral load (VL) measurements varied substantially among participants, with an overall median of 38.3 weeks (IQR 31.0, 42.4). As part of the intervention, 23 participants with VS and no known psycho-social issues consented and moved from the children’s hospital to the adult hospital clinic.

**Table 1:** Baseline socio-demographics and clinical characteristics of youth participants.

	Intervention (%)	Comparison (%)	Total (%)
**Participants**	137 (50.18)	136 (49.82)	273 (100.00)
**Mean age in years (range)**	19.12 (15–24)	19.10 (15–24)	19.11 (15–24)
**Age**			
15–19 years old	87 (63.50)	87 (63.97)	174 (63.74)
20–24 years old	50 (36.50)	49 (36.03)	99 (36.26)
**Sex**			
Female	82 (59.85)	80 (58.82)	162 (59.34)
Male	55 (40.15)	56 (41.18)	111 (40.66)
**Primary School**			
Did not complete primary school	20 (14.60)	11 (8.09)	31 (11.36)
Completed primary school	117 (85.40)	123 (90.44)	240 (87.91)
Missing	-	2 (1.47)	2 (0.73)
**How acquired HIV**			
From parents	97 (70.80)	101 (74.26)	198 (72.53)
Through sex	11 (8.03)	16 (11.76)	27 (9.89)
Another way	10 (7.30)	4 (2.94)	14 (5.13)
Don’t know	18 (13.14)	14 (10.29)	32 (11.72)
Missing	1 (0.73)	1 (0.74)	2 (0.73)
**Baseline VL suppression**			
**(< 1000 copies/ml)**	87 (63.50)	86 (63.24)	173 (63.37)
Missing	-	1 (0.74)	1 (0.37)
**Pre-ART CD4 count**			
median (25^th^, 75^th^)	285.5 (158–452)	280 (149–487)	284 (154–453)
0–349	61 (44.53)	57 (41.91)	118 (43.22)
350–499	12 (8.76)	14 (10.29)	26 (9.52)
500+	21 (15.33)	22 (16.18)	43 (15.75)
Missing	43 (31.39)	43 (31.62)	86 (31.50)
**Years on ART**			
median (25^th^, 75^th^)	7.19 (3.34–9.80)	7.45 (4.16–10.05)	7.28 (3.49–9.91)
<3 years	32 (23.36)	27 (19.85)	59 (21.61)
3–6 years	21 (15.33)	24 (17.65)	45 (16.48)
6+ years	83 (60.58)	83 (61.03)	166 (60.81)
Missing	1 (0.73)	2 (1.47)	3 (1.10)
**Weeks between baseline & midline**			
median (25^th^, 75^th^)	37.57 (30.00–41 71)	39.00 (32.71–43.43)	38.29 (31.00–42.43)

At midline 92% of participants allocated to the Intervention group (n = 126) and 85% of the Comparison group (n = 115) completed a survey and blood draw. Results from the primary analyses on VS, ART adherence treatment gap, and internalized stigma are presented below. No known unintended harmful effects of the intervention were found.

### Viral suppression

**Fig 2:**
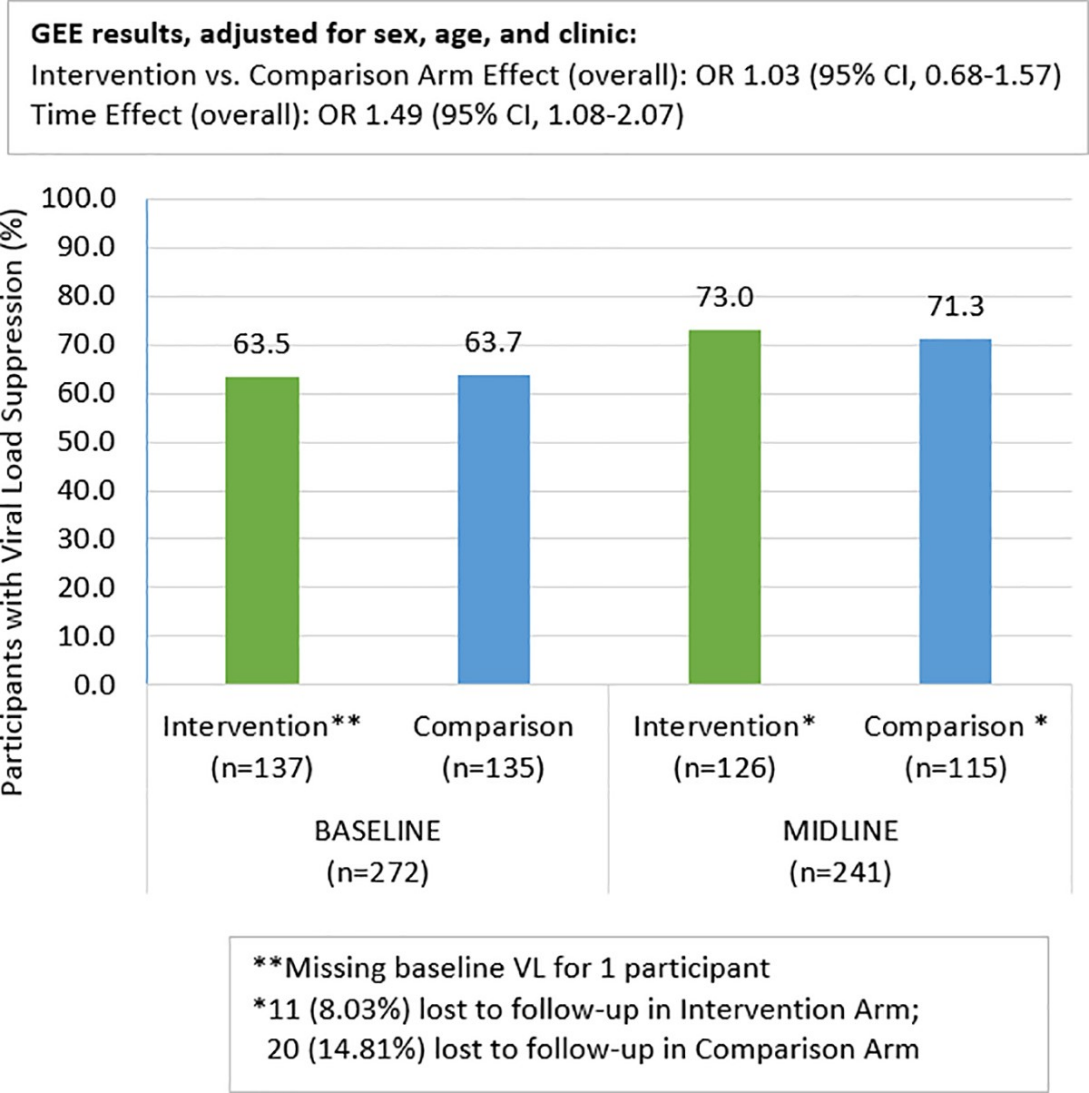
Viral suppression at baseline and midline by study arm.

Fig 2 shows the counts and percentages of participants with VS at baseline and midline by study arm. At midline, 20 participants in the comparison arm and 11 in the intervention arm were lost to follow-up (two youth from each study arm died before starting intervention activities, none of whom were suppressed at baseline). VS was similar in both arms and increased from baseline values of 63.7% and 63.5% in the comparison and intervention arms respectively, to 71.3% and 73.0% at midline. The results of the GEE model confirmed that the odds of VS was similar between arms [Odds ratio (OR):1.03, 95% Confidence Interval (CI):0.68, 1.57]. The GEE model also showed a significant increase in VS between baseline and midline in both arms [OR: 1.49, 95% CI:1.08, 2.07], and no evidence of an interaction between study arm and time. As such, the final primary model for this analysis did not include the interaction term. The inverse probability weighted estimate had similar results: The odds of VS was similar between arms [OR 1.02, 95% CI: 0.66, 1.55], and there was a significant increase in VS between baseline and midline in both arms [OR 1.52, 95% CI: 1.09, 2.10], and no evidence of an interaction between study arm and time.

The results also show that there was no evidence of a mediation effect of the length of time between baseline and midline VL measurements [indirect effect: -.034, 95% CI: -0.12, 0.05] and there was no significant partial effect of study arm on the time between baseline and midline [partial effect: -.2,8 95% CI: -0.68, 0.11]

### Viral suppression analysis stratified by treatment site type (pediatric versus adult)

**Fig 3:**
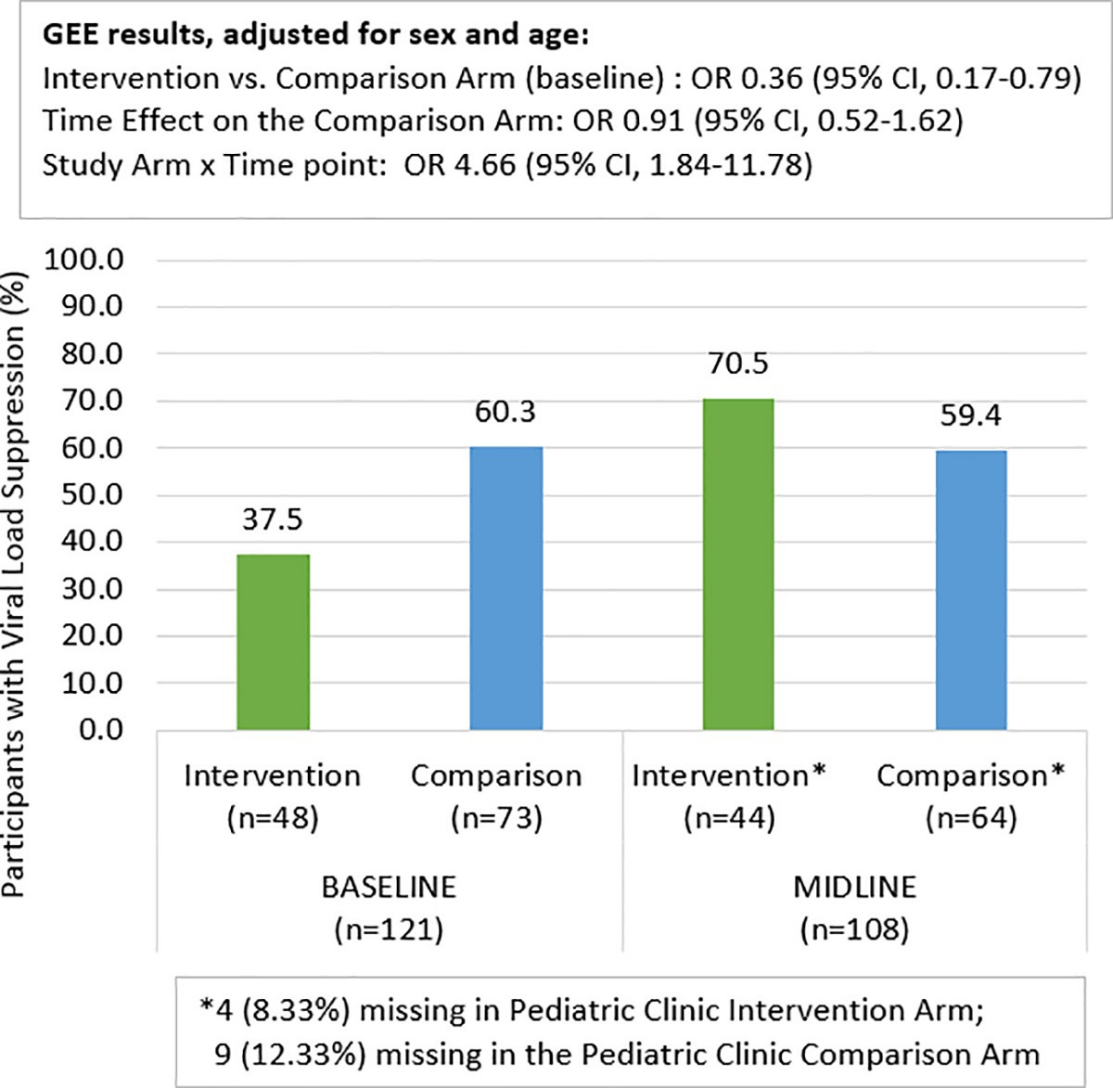
Viral suppression at baseline and midline: Pediatric clinic only.

**Fig 4:**
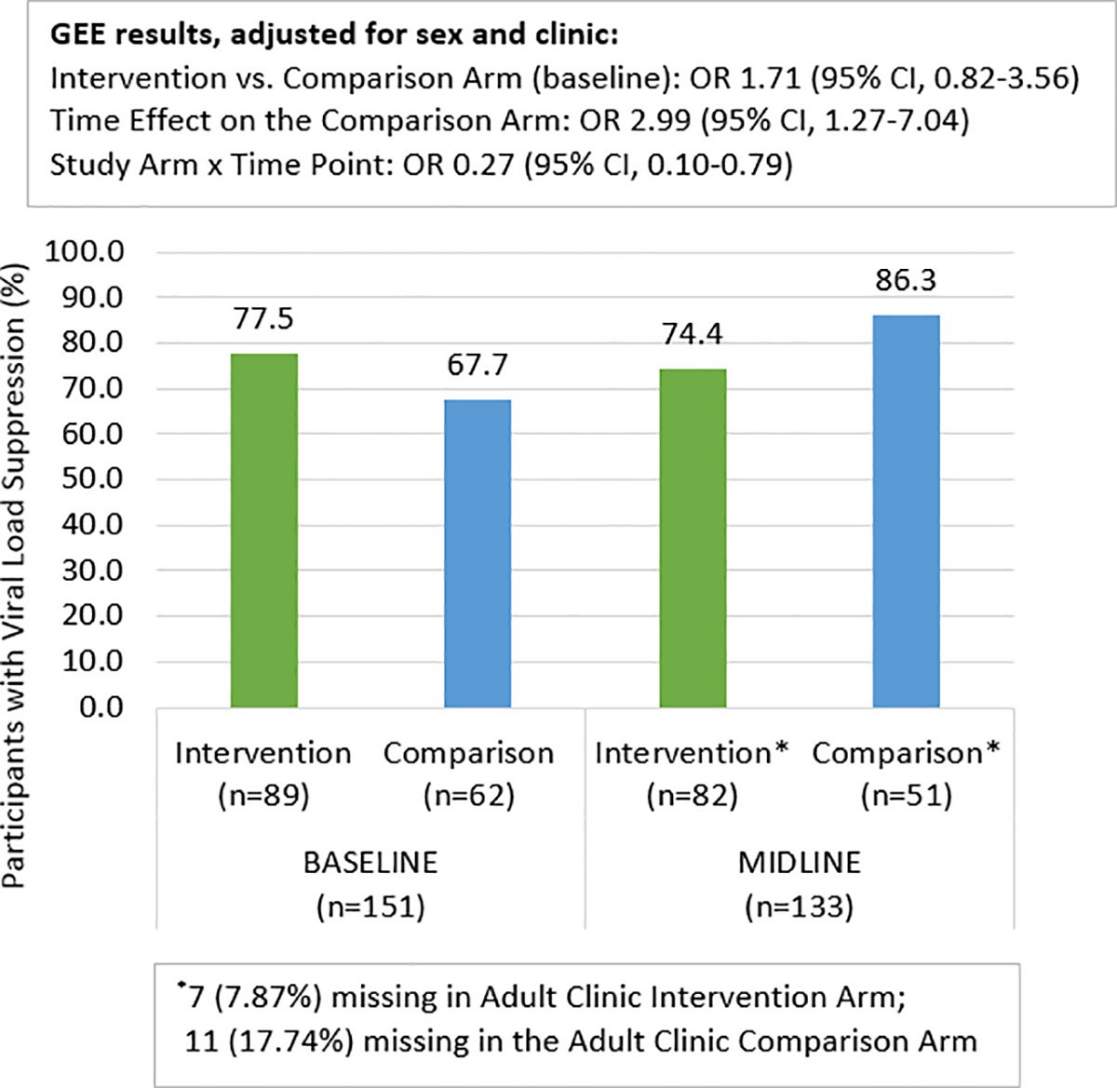
Viral suppression at baseline and midline: Adult clinics only.

Figs 3 and 4 show VS at baseline and midline by study arm and by the type of site (pediatric or adult) where the participant received the intervention. In the children’s hospital, VS at baseline was 60.3% and 37.5% for the comparison and intervention arms respectively. The comparison arm did not show a change by midline (59.4% suppressed) while the intervention arm improved to 70.5%. The GEE model for the pediatric site confirmed that at baseline, the intervention arm had a significantly lower level of suppression than the comparison arm [OR:0.36, 95% CI:0.17,0.79]. Among comparison arm participants, the odds of VS did not increase at midline relative to baseline [OR:0.91, 95% CI:0.52,1.62]. There was a significant arm by time interaction with the intervention arm participants experiencing a relative increase in the odds of VS by a factor of 4.7 relative to comparison arm [interaction term OR:4.66, 95% CI:1.84,11.78].

In the adult clinic settings, VS at baseline was 67.7% and 77.5% for the comparison and intervention arms, respectively. The comparison arm improved to 86.3% suppressed by midline while the intervention arm changed slightly to 74.4%. The GEE model for the adult sites showed that among the comparison arm participants, time had a significant effect so that the odds of VS at midline were three times higher than at baseline [OR:2.99, 95% CI:1.27,7.04]. Furthermore, among the intervention arm participants, there was a significant relative reduction in the odds of VS by midline compared to the comparison arm participants [interaction OR:0.27, 95% CI:0.10, 0.79].

### ART adherence

**Fig 5:**
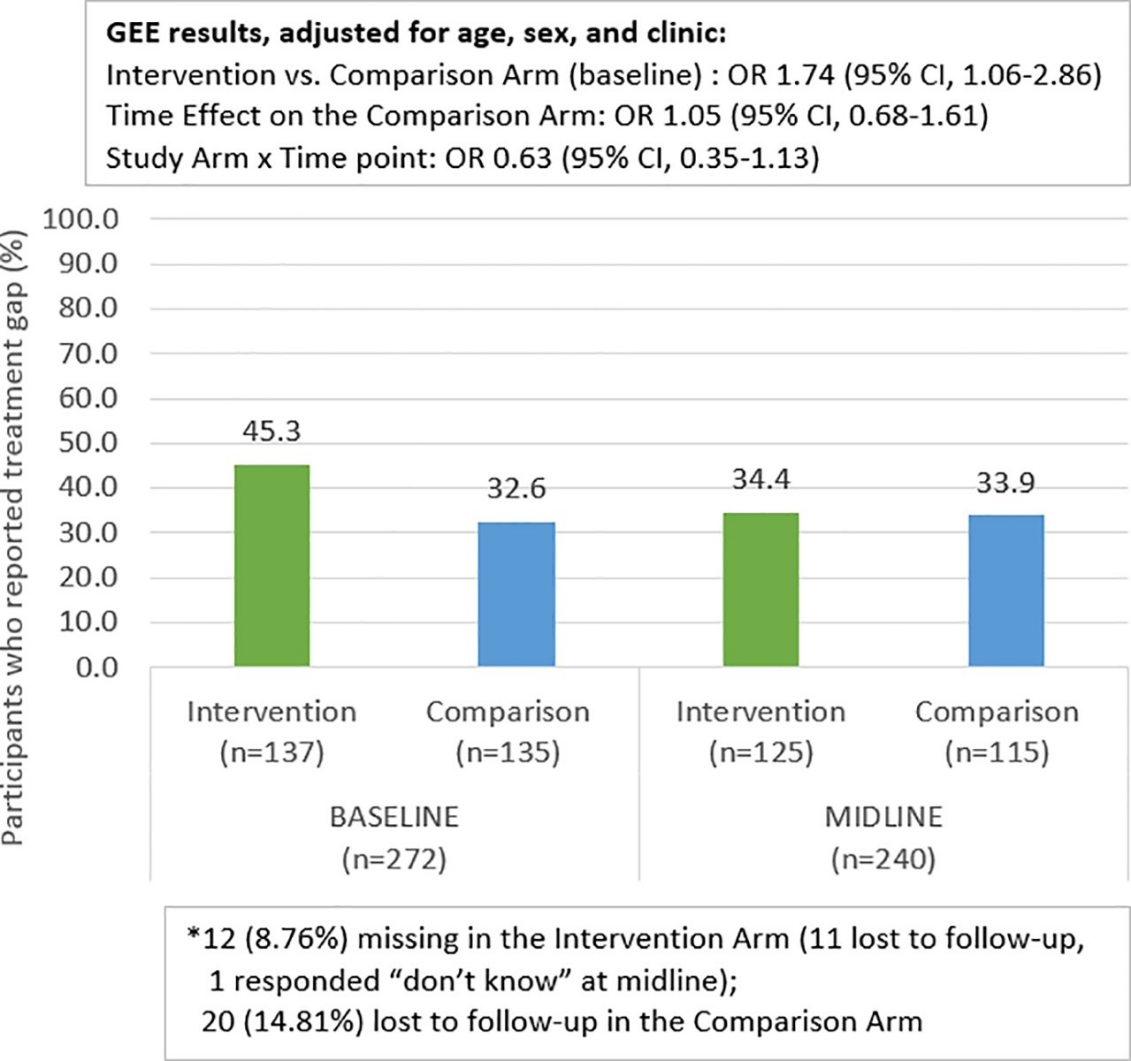
ART Adherence Treatment Gap (48 hour or more in the past 3 months) at baseline and midline by study arm.

Fig 5 shows that among the comparison arm participants 44 (32.6%) at baseline, and 39 (33.9%) at midline, reported an ART adherence treatment gap of 48 consecutive hours or more in the past three months. Among the intervention participants, 62 (45.3%) at baseline, and 43 (34.4%), at midline reported an ART adherence treatment gap. The GEE model indicates that the intervention had significantly higher odds of treatment gap than the comparison arm at baseline [OR:1.74, 95% CI:1.06, 2.86]. Having a treatment gap did not change for the comparison group between baseline and midline [OR:1.05, 95% CI: 0.68, 1.61], but there was a notable relative change for the participants in the intervention arm [OR interaction term: 0.63, 95% CI: 0.35, 1.13]

### Internalized stigma: Feelings of guilt, shame, worthlessness

**Fig 6:**
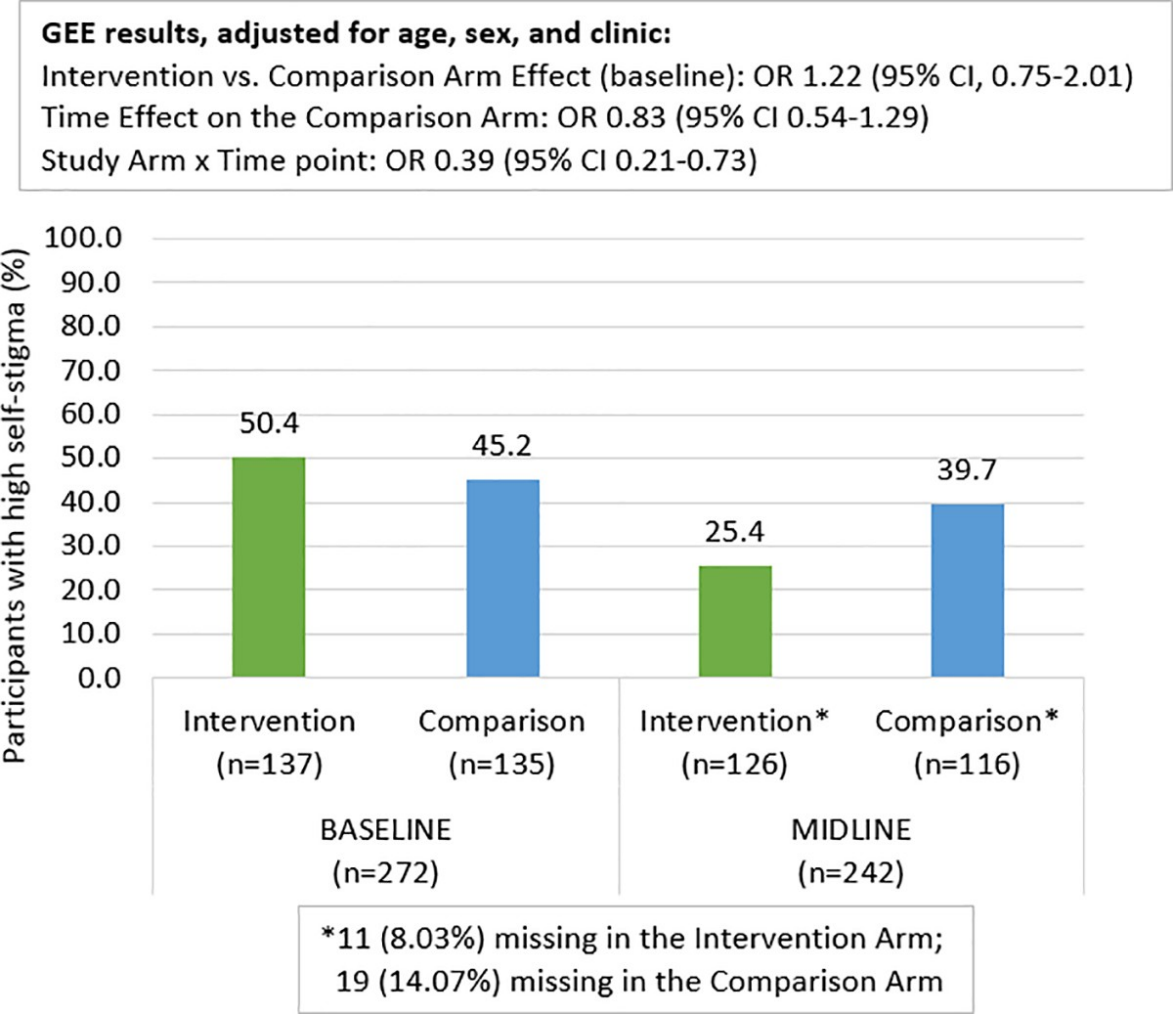
Self-Stigma at baseline and midline by study arm.

The Cronbach’s alpha for the three items of internalized stigma was 0.74 at baseline and 0.73 at midline. Fig 6 shows that in the comparison arm, 61 (45.2%) participants at baseline and 46 (39.7%) participants at midline reported at least two of these feelings. In the intervention arm, these feelings were prevalent among 69 (50.4%) participants at baseline which was reduced to 32 (25.4%) at midline, suggesting a potential time by arm interaction term. The GEE model shows that there was no statistically significant difference between baseline and midline among comparison participants [OR:0.83, 95% CI:0.54,1.29], but there was evidence of an interaction so that for intervention arm participants, the odds of having these feelings were significantly reduced by a factor of 0.39 relative to the reduction in the comparison arm [interaction terms OR:0.39, 95% CI :0.21,0.7].

#### Exposure

Among the 126 intervention arm participants with a midline viral load test, 120 (95%) attended an orientation meeting, and the majority (93 [73.8%]) attended five or six one-on-one meetings with a YPM (Table 2). Given a lack of variability in attendance and the small numbers when stratified by VS, a further analysis to explore the potential relationship between VS and Project YES! exposure to one-on-one meetings was not conducted. Almost half (57 [45.2%]) of the participants attended five or six group meetings. There was no significant association between attending at least five youth group meetings and VS at midline (Chi-squared p-value = 0.66). In terms of caregiver support, 59 (47%) of youth had at least one caregiver who attended one Project YES! session. There was no significant association between having a caregiver attend at least one of the meetings and VS at midline (Chi-squared p-value = 0.14). We evaluated baseline characteristics to see if they were associated with caregiver attendance and found that younger participants (15–19 years old) were more likely to have a caregiver attend versus the older (20–24 years old) participants (51.7% vs 28% p-value = 0.007). There was no evidence of an association with sex nor intervention site.

**Table 2:** Exposure to the Project YES! and viral load suppression at follow-up among intervention arm participants (n = 126)*.

	VL Suppression (%)	Non-VL suppression (%)	Total
**Orientation mtg**			
Yes	87 (94.6%)	33 (97.1%)	120 (95.2%)
No	5 (5.4%)	1 (2.9%)	6 (4.8%)
**One-on-one YPM mtgs**			
0–4	25 (27.2%)	8 (23.5%)	33 (26.1%)
5–6	67 (72.8%)	26 (76.5%)	93 (73.8%)
**Group YPM mtgs**			
0–4	54 (58.7%)	15 (44.1%)	69 (54.8%)
5–6	38 (41.3%)	19 (55.9%)	57 (45.2%)
**Any caregiver engagement**			
Yes	42 (45.7%)	17 (50.0%)	59 (46.8%)
No	50 (54.3%)	17 (50.0%)	67 (53.2%)

*11 lost to follow-up in the Intervention Arm

We also assessed potential contamination between the two study arms. Overall, across all the clinics, less than 5% (N = 12, 4.96%) of participants at the 6-month midline reported they had talked with youth in the other arm about session content (5 intervention group participants and 7 comparison group participants). The topics most frequently discussed across both study arms were “adherence or taking your ART drugs” (N = 9, 3.72%) followed by “Taking care of your own health [self-management]” (n = 7, 2.89%) and “viral load test results and how to suppress HIV in the blood” (n = 7, 2.89%).

## Discussion

YPMs, when well trained and paid, are a feasible and effective way to reduce internalized stigma and achieve VS among AYA living with HIV. Through a process of capacity building, these young people, most of whom had never held formal employment before, were able to gain the skills and self-confidence to share their experiences to mentor AYA and to become valued and skilled clinic employees.

In this context, mentoring is substantially different from being a peer educator. While providing accurate information as a trustworthy peer source is a critical component of both educating and mentoring, mentoring also encompasses active role-modeling, listening, and problem-solving based on the shared experience of living with HIV. It is this shared experience coupled with a clear understanding of boundaries that allowed the Project YES! YPMs to serve as empowered role models, demonstrating the behaviors of positive living and HIV self-management. Boundaries were particularly important because the YPMs were not trained to be counselors or to attempt to resolve certain complex issues AYA may face, such as experiences of violence and/or suicide ideation. Knowing one’s role, boundaries, and when to refer AYA clients to clinical staff were important aspects of the YPM capacity-building process. Overall, the YPMs took ownership of and shaped program delivery, becoming the experts. This approach complements and builds upon existing support in Zambia for adolescent engagement in HIV care as illustrated by the Ministry of Health’s Facilitator’s Guide for Adolescent Peer Educators [44]. The Project YES! youth-driven process was also well received, as indicated by the high uptake of the one-on-one meetings and represents an important approach to addressing the needs of AYA living with HIV.

A main finding of Project YES! was the significant decrease in internalized stigma experienced by intervention arm participants across all study sites. AYA are in a developmental stage when they are “developing and consolidating their sense of self” [45], and the impact of internalized stigma among this age group is not well studied [46]. In a systematic review of stigma-reduction interventions in low- and middle-income countries, the authors found only one study that focused on youth [46, 47]. This study used motivational interviewing to change sexual risk behaviors and alcohol use among Thai youth and did not find evidence of an impact on internalized stigma. Project YES! addresses this distinct gap in knowledge by providing critical intervention data on how to reduce internalized stigma. These results further support a growing body of literature on the relationship between internalized stigma, identity, developmental stage and chronic illness outcomes among AYA [48–50].

Another key finding was the relative increase of 4.7 in VS among AYA intervention versus comparison participants in the pediatric clinic at the children’s hospital. This finding provides important evidence on the impact YPMs have when working in collaboration with AYA clients, clinic staff and caregivers. Evidence of this effect, however, was not found in the adult setting. In the adult setting, the intervention arm had significantly greater VS at baseline than the comparison arm, possibly due in part to the fact that 23 virally suppressed participants from the children’s hospital clinic were transitioned to the adult hospital before the start of study activities as part of the intervention and included in the adult clinic population for analysis. This higher level of VS at baseline meant less opportunity for change over time for participants in adult clinics. In addition, the adult setting comparison arm had twice the loss to follow-up as the adult setting intervention arm, while the loss to follow-up was minimal in both study arms in the pediatric setting. These research findings and biases reinforce the need to further study AYA experiences in adult HIV clinic settings and to assess if reductions in internalized stigma lead to increased VS over time. The large increase in VS found among AYA in the pediatric clinic, however, is clear and this is one of the only studies that provides concrete evidence of program impact on increasing VS in this age group. The fact that the intervention arm participants in the pediatric clinic had higher levels of viral failure at baseline (given the transition of those with viral suppression to adult settings) indicates the potential role Project YES! may have in the provision of differentiated care focusing on AYA who are experiencing challenges with their viral status. These findings have direct implications for programing and the need to incorporate peer mentors into HIV clinic services in pediatric settings.

While the study did not find a significant relative reduction in ART adherence treatment gaps among intervention versus comparison participants, we did see an overall decrease in ART adherence treatment gaps from 45.3% to 34.4% in the intervention arm. A limitation of this study is the adherence measures were self-reported and may reflect social desirability bias. Newer technologies, like point of care (POC) urine test to measure adherence to Tenofovir-based regimens [51–53], will help to evaluate how programs can improve ART adherence in future studies.

Other limitations to consider include the individual randomization within clinics that may have resulted in the comparison arm participants experiencing changes in usual care given that several clinic staff, during in-depth interviews, reported that participating in Project YES! changed the way they see and interact with all of their AYA patients [54]. Such changes may have influenced HIV outcomes among comparison arm participants as they were attended to by the same HCP as the intervention participants [55]. Purposive sampling of study sites and consecutive sampling of study participants may also introduce selection bias and limit generalizability of the study findings.

Overall, the Project YES! results establish YPMs as a valuable, underutilized resource to support AYA living with HIV in an overburdened health care system. Peer mentoring approaches have worked with other populations [29, 30], and contributes to Zambia’s commitment to AYA engagement [44] by training youth to serve not only as peer educators but also as mentors and role models for their HIV-positive peers. Future research will focus on issues of scale-up of YPM integration into clinical care using implementation science strategies.

## Conclusions

Project YES! provides a feasible and effective clinic-based approach to engage AYA and improve their HIV-related outcomes. The key intervention component was implemented and led by well-trained and paid youth, exemplifying how a virtually untapped resource in the HIV epidemic–young people–can successfully engage and shape HIV outcomes among AYA.

## Supporting information

S1 Checklist CONSORT 2010 checklist of information to include when reporting a randomised trial*. (DOC)

S1 Research Plan (PDF)
