# Correction: Project YES! Youth Engaging for Success: A randomized controlled trial assessing the impact of a clinic-based peer mentoring program on viral suppression, adherence and internalized stigma among HIV-positive youth (15-24 years) in Ndola, Zambia

**DOI:** 10.1371/journal.pone.0232488

**Published:** 2020-04-23

**Authors:** Julie A. Denison, Virginia M. Burke, Sam Miti, Bareng A. S. Nonyane, Christiana Frimpong, Katherine G. Merrill, Elizabeth A. Abrams, Jonathan K. Mwansa

In the Funding section, the grant number from the funder Project SOAR is listed incorrectly. The correct grant number is: AID-OAA-A-14-00060.
