# Supplementary material for: Project YES! Youth Engaging for Success: A randomized controlled trial assessing the impact of a clinic-based peer mentoring program on viral suppression, adherence and internalized stigma among HIV-positive youth (15-24 years) in Ndola, Zambia
Source: PLoS One. 2020 Apr 2;15(4):e0230703. doi: 10.1371/journal.pone.0230703 (PMC7117673; doi:10.1371/journal.pone.0230703)
Supplement: S1 Research Plan — (PDF) [file pone.0230703.s002.pdf]

JHSPH IRB Research Plan for New Data Collection

PI Name: **Julie Denison**
Study Title: **Transitioning Adolescents to HIV Self-Management in Zambia (Known as: Project YES: Youth Engaging for Success)**
IRB No.: IRB00007870
PI Version No. / Date: 5 / September 7, 2018

I. <u>Aims of the Study</u>: Describe the aims/objectives of the research and/or the project's research questions or hypotheses.

The specific objectives of the study are to:

AIM 1) Assess the impact of a peer mentoring approach to implementing the AIDSTAR-One toolkit on viral suppression and other HIV-related outcomes such as retention in care and adherence to cART, among transitioning youth in different care facility settings (e.g., pediatric hospital, adult hospital, and primary care facilities). Hypothesis: Youth in the intervention arm will experience greater HIV viral suppression at the end of six months of the intervention than youth in the comparison arm.

> AIM 1a) Conduct a stratified analysis to examine the impact of a peer mentoring approach separately among: a) the 144 youth participants (15-24) transitioning out of an adolescent clinic into an adult clinic; and b) among 144 youth participants (15-24) who are already attending adult care. This will be a sub-analysis of the larger study to determine the effects of the intervention among these two distinct groups of adolescents and youth.

AIM 2) Examine the experiences of youth and their families, health care providers, peer mentors, and study staff with the peer-mentoring/toolkit program: This objective will be achieved by conducting about 80 qualitative in-depth interviews (IDIs) with the youth participants, as well as about 30 IDIs with their family caregivers. We will also interview the health care providers and peer mentors involved in implementing the program, estimated to be around 10 and 12 people respectively. We will further interview study staff involved in implementing the referral process established for the study (see Section 7.4), estimated to be around 6 people. Out of the 80 youth who will be interviewed, half will be sampled on viral load (VL) test results (e.g. have VL failure at baseline and suppression at midline) and half will be sampled based on VL test results and varying experiences of violence. Interviews with the youth, caregivers, healthcare providers, and peer mentors will provide data on the positive and negative experiences these four groups had with the intervention and the mechanisms believed to support or hinder adolescents' viral suppression. By sampling half of the youth specifically on violence experiences, we will also investigate how youth's experiences of violence victimization may relate to their HIV self-management and how the intervention has helped youth deal with these violent experiences, including through the referral procedures established for the study. Interviews with the study staff will offer additional insight into the effectiveness of the referral procedures put in place to support youth with experiences of violence victimization, mental health issues, and other concerns about their wellbeing. These data will contextualize the quantitative data findings and ultimately inform and strengthen the intervention approach.

II. <u>Background and Rationale</u>: Explain why this study is being done. Summarize briefly what is already known about the issue and reference previously published research, if relevant.

While the literature on pediatric chronic illnesses often emphasizes the needs of adolescents transitioning from pediatric to adult care, there is no published literature on interventions to support HIV-positive adolescents transitioning to adult care and/or HIV self-management in sub-Saharan Africa (SSA). Similarly, there is virtually no data on how to best engage families and peers to support youth (YLHIV) with their care needs in SSA, despite the known impact families and peers have on adolescent health (1-3). For example, a 2015 review found only 14 studies on adolescents living with HIV (ALHIV) transitioning to adult care, all of which were conducted in the US or UK and the majority of which were qualitative studies with sample sizes of 50

participants or fewer (4). A 2016 systematic review examined the literature to assess the effectiveness of self-management interventions for young people across chronic illnesses. Out of 42 randomized controlled trials included in the review, none were conducted in SSA. The authors note that most interventions focused on the medical aspects of self-management rather than psycho-social issues (5). The authors also discuss the potential role for online peer support (5). These findings correspond with other articles that define three specific aspects of self-management: medical management (e.g., treatment adherence), role management (e.g., social participation), and emotional or identity management (e.g., feelings, stigma) (6, 7).

Despite this lack of evidence on how to support youth, the need remains great. Preliminary analysis of routine viral load (VL) testing conducted among ALHIV clinic attendees at the Arthur Davison Children's Hospital (ADCH) in Ndola, Zambia revealed that around 50% of ALHIV have viral failure defined as 1000 copies/ml or higher. This staggering percentage underscores the urgent need for assistance with care for this population.

This study will address this gap by testing a peer-mentoring program to implement the AIDSTAR-One toolkit for transition (https://aidsfree.usaid.gov/sites/default/files/final_alhivtoolkit_web.pdf). AIDSTAR-One developed this toolkit as a resource for health care providers to support youth transitioning to HIV self-management as well as their caregivers. The current version, released in 2014, includes a transition readiness checklist designed for health care providers to monitor an adolescent's ability to self-manage his/her HIV. The toolkit also has corresponding modules that cover 10 subject areas deemed critical for youth's successful HIV self-management, including psychosocial development, mental health, sexual and reproductive health, alcohol and substance abuse, beneficial disclosure and clinical considerations.

While there have been no large scale implementations of this toolkit, the initial version was piloted in Kenya in 2012 "to inform final adaptations prior to dissemination" (8) A survey conducted among 17 health care and community care providers in Kenya who were trained on the AIDSTAR-ONE toolkit, found that a majority were satisfied with the kit. A key concern expressed, however, was the time needed to implement the toolkit approach in settings where providers are overburdened. A subsequent key recommendation made by providers during this pilot evaluation was to expand the use of the toolkit to community-based providers, including specifically peer counselors or mentors. Peer mentors, especially those who are trained well and given paid positions, have been effective in other settings, such as the Mothers to Mothers program in South Africa (9) or the Restless Development program in Zambia (10). In South Africa, paid mentors who are mothers themselves and had undergone prevention of mother to child HIV transmission (PMTCT) learned concrete employable skills and were integrated into the health care system to mentor women undergoing PMTCT. In Zambia, a youth intervention was conducted that placed young adults (18 to 24 years) in government schools to teach a Ministry of Education life skills course. Again, these youth mentors were paid, well trained, and given a position within the system. Results from an evaluation found that students in the schools with youth mentors had more HIV and reproductive health knowledge, and lower levels of stigma and sexual risk-taking behaviors, than their peers in schools without youth mentors (10). In this study, peer mentors will be integrated into the health care system to implement the toolkit.

This study will further explore the potential integration of violence-related content into the intervention. Despite limited existing literature on violence victimization among HIV-positive youth in SSA (11-14), studies among adults in SSA and elsewhere have documented negative impacts of violence victimization on HIV disclosure (15-18), linkage to care (19, 20), and engagement and retention in care ((20, 21). A deeper understanding is needed of the types of violence experienced by youth, the influence of such violence on youth's HIV care and treatment, and the potential role of the adapted AIDSTAR-One toolkit for transition in addressing such violence.

The overall goal of this study is to test a peer-mentoring approach to implementing the AIDSTAR-One toolkit to improve HIV-related outcomes, including viral suppression, among youth as they transition to, and engage in, self-management and adult HIV care and treatment.

III. <u>Study Design:</u>

A. Provide an overview of your study design and methods. The study design must relate to your stated aims/objectives. Details will be requested later. If your study also involves analysis of existing data, please complete Section XI, “Secondary Data Analysis of Existing Data” in the last part of this research plan. If your study ONLY involves analysis of existing data, please use the research plan template for secondary data analysis (JHSPH IRB Research Plan for <u>Secondary Data Analysis of Existing Data/Specimens</u>).

The study is a randomized controlled trial at the individual level that uses a stepped wedge design to assess the peer-mentoring/AIDSTAR-One toolkit intervention. The study team will test a six-month peer-mentoring program among 144 YLHIV in an intervention arm compared to 144 YLHIV in a comparison arm. Participants for the RCT will be consecutively selected from two hospitals -- Arthur Davison Children’s Hospital (ADCH) and Ndola Teaching Hospital (NTH) – and two primary care clinics (PCCs) – Twapia Clinic and Lubuto Clinic. The participating facilities were purposively selected because they represent different models of care. ADCH is a hospital for children and serves older youth who should ultimately transition to adult care. In contrast, NTH and the two primary care clinics are essentially adult HIV care settings. While they were not designed to initially serve HIV positive adolescents, they continue to experience a growth in the number of enrolled adolescents. The PCCs were purposively selected based on their high ALHIV patient populations, designated ART center status, and proximity to other study sites.

After the completion of the initial 6-month intervention and the subsequent 6-month follow-up assessment among participants in both arms, the primary intervention group will enter a maintenance phase and the comparison group will receive the intervention for six months. Assessments, including a survey and a blood draw for VL testing, will occur at baseline, at the end of the first six months, and at the end of 12 months (see Figure 1 under study implementation). Resistance testing will also be conducted at baseline for those participants who have a VL failure, defined as 1000 copies/ml or higher. Resistance testing is critical for this study as the primary outcome is viral suppression, something that will not occur if a participant has a resistant strain and is not on the correct treatment, no matter how adherent s/he may be. Routine VL results from among ADCH patients reveals that approximately 50% of adolescents have VL failure.[1] Based on that data, we estimated that 50% of the 288 participants in this research (n=144) will have VL failure and need resistance testing.

At the end of the initial 6-month intervention, we will begin conducting qualitative in-depth interviews with participants, their caregivers when possible, and the health care providers, peer mentors, and study staff involved in delivering the program. These data will provide contextual details and experiences to help interpret the study findings and determine what aspects of the intervention were valued.

B. Provide a sample size and a justification as to how you arrived at that number. If you use screening procedures to arrive at a final sample a table may be helpful.

AIM 1 Calculations

The sample size calculation is based on recent data on viral load suppression. This intervention is designed to support adolescents to manage their HIV as they transition into adulthood to achieve healthy outcomes, with viral suppression being the strongest outcome measure that is feasible. Viral suppression is a key health outcome we aim to achieve and maintain through the transition process and is of particular importance as ADCH program data has indicated that only 50% of 15-19 year olds are virally suppressed, with 50% having viral loads of 1,000 copies/ml or greater. With group sample sizes of 144, we will have over 85% power to detect a difference of 20% (an increase from 50% to

[1] Data are from ADCH’s routing VL screening, which is being conducted among adolescents who had been on cART for a minimum of 6 months, although many have been on cART for years.

70%) between the proportions virally suppressed in the intervention versus the control arms at the six-month data collection point, with Type 1 error of 5%, and 20% loss to follow-up. The team will also explore differences in self-reported adherence among the two study arms, with adherence assessed using a 30-day and 48-hour treatment gap measures that we have used in our previous research. Adherence is a proxy for achieving viral suppression. Adherence is also one of the toolkit module topics and maintaining adherence is a criterion for transition in the readiness to transition checklist.

Sub-AIM 1A Calculations
For sub-Aim 1a, the stratified analysis, the sample size of 144 youths from ADCH who are transitioning to adult care will achieve 80% power to detect a 25% difference in viral suppression among the roughly 70 youths in the intervention versus the comparison arm (an increase from 50% to 75%). The same calculation holds true for the analysis examining the impact among the 144 adolescents who are already attending adult care at three other study sites.

AIM 2 Calculations
By conducting IDIs with about 40 youth sampled on VL test result (e.g. have VL failure at baseline and suppression at midline) and 40 sampled based on VL test result and varying experiences of violence, the IDIs should reach saturation on key themes while being able to stratify responses by sex, age, whether the youth transitioned, and clinical setting

The caregiver sample size will be about 30. With 30 IDIs, we should reach saturation across caregivers with differing degrees of involvement in the program. In addition, all health providers (estimated to be 10) who have a significant involvement with the intervention, all peer mentors (estimated to be 12), and all study staff (estimated to be 6) who have a significant involvement in the implementation of the referral process will be invited to participate in an IDI to capture the full experience of intervention implementation from these perspectives.

IV. **<u>Participants</u>:**

Describe the study participants and the population from which they will be drawn. Specify the inclusion and exclusion criteria. If you plan to include children, note their ages and whether you will include children in foster care. Note if the participants are particularly vulnerable in terms of cognitive limitations, education, legal migration status, incarceration, poverty, or some combination of factors.

A. **Inclusion Criteria:**

AIM 1:
To be eligible to participate in the study, a participant must be:

- In the age range of 15-24 years at ADCH and 15-24 years at NTH and the PCCs),
- Aware of her/his HIV status
- On cART for at least six months
- Speaks Bemba or English
- Not planning to move out of the district in the next 18 months, and planning to be available to attend study activities over the next 18-months, as needed.

AIM 2:
Youth participants will be eligible to participate in an in-depth interview if they were enrolled in AIM 1. Purposive sampling strategy will be used based on key characteristics mentioned. We will also seek to recruit youth from the different clinic settings and who have different levels of caregiver involvement (e.g. whether or not they had a caregiver who participated in study activities).

Youth who participate in IDIs as part of AIM 2 (n=40) will be asked if they have a caregiver whom they want to invite and the study staff may contact to invite for an IDI. Youth do not need to have a caregiver to invite to participate in these AIM 2 interviews. Study staff may also directly contact caregivers of youth participants from AIM 1 to invite them for an in-depth interview.

All health care providers and peer mentors who implemented the program, as well as study staff who implemented the referral process, will also be invited to participate in an IDI.

B. **Exclusion Criteria:**

Exclusion criteria for youth include being too sick to participate, attending boarding school, having a sibling already enrolled in the study (one youth per household), or having participated in the recent NIH-funded R34 Positive Connections intervention.

V. Study Procedures:

In this section, provide details of your procedures, particularly as they relate to human subjects. If this is a multi-center study, make the role of JHSPH clear. If the JHSPH will serve as **data coordinating center**, indicate in the sections below which procedures JHSPH will not be performing. Additional information regarding data coordinating centers is requested in a later section. If your study will develop in phases, address each item below by phase.

A. **Recruitment Process:**

1. Describe how you will identify, approach, and inform potential participants about your study. Include details about who will perform these activities and what their qualifications are.

AIM 1:

We will recruit participants consecutively as they attend the HIV care and treatment clinics, aiming to achieve diverse enrollment based on sex and age. When possible, SMART Care appointment lists will be made available by clinic staff to assist the study team in anticipating the numbers of potentially eligible youth scheduled to be seen at each study site (SMART Care is the electronic medical record system). Trained clinic staff and study staff members will work in tandem to recruit eligible participants using a recruitment script. Clinic staff will have access to each patient's medical chart and will work to ensure that patients who fall within the age range and are aware of their HIV-positive status are approached. They will then refer interested youth to a study staff member. The study team has had success recruiting adolescents using these methods in their previous research in Zambia. Once enrolled the study team member will place a sticker on a back page of the youth's medical file (a page not typically used by clinic staff and not visible from outside the folder) to indicate participation in the study. This sticker will help ensure the study team does not enroll the same person twice and also ensure that the test results are placed in the correct file. Ultimately, we plan to enroll an estimated 144 participants from ADCH, a pediatric clinical setting, and 144 from three adult settings: NTH, Lubuto Clinic, and Twapia Clinic.

AIM 2:

We plan to recruit about 80 youth participants purposively from our AIM 1 study population. For 40 interviews, our priority will be to achieve diversity in the youth's VL test results. For the remaining 40 interviews, we will aim to achieve diversity in youth's VL test results and in their experiences of violence victimization, as reported on the baseline surveys. Across all 80 interviews, we will further seek to achieve diverse enrollment based on key characteristics of the youth such as sex, age and clinical setting. Additionally, we plan to interview about 30 caregivers, as well as the health care providers, peer mentors, and study staff who implemented the program (estimated to be 10 health care providers, 12 peer mentors, and 6 study staff who supported the referral process).

2. Address any privacy issues associated with recruitment. If recruitment itself may put potential participants at risk (if study topic is sensitive, or study population may be stigmatized), explain how you will minimize these risks.

   Health care providers will only refer youth who are aware they are living with HIV to study staff for recruitment. Study staff will talk with interested youth in a private space in or near the health facility at which they are seeking care.

B. **<u>Consent Process</u>**:

1. Describe the following details about obtaining informed consent from study participants. If a screening process precedes study enrollment, also describe the consent for screening.

   a. Who will obtain informed consent, and their qualifications:

      All study staff who obtain informed consent will be trained on the ethical conduct of the study including the informed consent process. When possible, we will hire individuals to collect data who have previously worked on similar research with this population. This staff includes the data collectors, the research assistant and coordinator. Christine Jere, the Research Coordinator in Zambia, has a bachelor's degree in development studies from the University of Zambia, a transcript diploma in social work, and experience working with the PI on previous research with youth living with HIV. Lindy Imboela Mbando has a diploma in social work from the University of Zambia and has worked with the PI on previous research with youth living with HIV. Christy Frimpong, the Program Implementation Manager, has a bachelor's degree in Agric Science, focusing in Animal Science from the University of Ghana and a Master's degree in Public Health, focusing in Epidemiology and Biostatistics from University of Zambia. In addition, Virginia Burke, the overall study coordinator, who has an M.S.P.H., and Kate Gannett, a doctoral student in the SBI IH program, may conduct in-depth interviews for AIM 2.

   b. How, where, and when the consent discussion(s) will occur:

      Study staff will complete the informed consent process in a private area in or near each of the clinic study sites. Youth participants will be consented at enrollment for AIM 1 & 2 activities. Caregivers will be consented the first time they attend a study-related event (AIM 1) and before their in-depth interview (AIM 2), if applicable. Healthcare providers, peer mentors, and study staff will be consented before their in-depth interview (AIM 2).

      Consent will take place in a private space in or near the health clinic. The study staff will read the informed consent/assent forms aloud with the potential participant and ask questions about risks and benefits to ensure comprehension. Original signed informed consent forms for each participant will be kept in a locked place.

   c. The process you will use to determine whether a potential participant meets eligibility criteria:

      AIM 1:
      Initial eligibility will be determined by the health care staff based on their regular clinic procedures, whereby they pull the person's medical chart when they arrive at the clinic. If the youth is eligible and interested, the health care providers will refer the young person to a study staff member who will confirm eligibility, explain the study in more detail and go through an informed consent process. Youth will be asked to identify potential caregivers for participation.

      AIM 2:
      All youth who participated in AIM 1 are eligible to participate in an in-depth interview. Caregivers are eligible for an in-depth interview if they have a youth who is participating in an in-depth

interview. All health care providers and peer mentors involved in the implementing the program, as well as study staff who implemented the referral process, are eligible to participate in an in-depth interview.

d. Whether you will obtain a signature from the participant or will use an oral consent process:

   We will obtain a signature or, if the participant is illiterate, a thumb print from each participant. A copy of the informed consent form in Bemba or English will be offered to each participant**.**

e. Whether you will obtain a legally authorized representative's signature for adults lacking capacity:

   N/A

f. If children are included in the study, if and how you will obtain assent from them:

   In Zambia, the 2013 Health Services Research Act (Act number 2 of 2013) defines a minor as anyone younger than age 18, and parental consent is required for all minors to participate in research. Based on this Act, the team will require written parental/caregiver adult consent and written youth assent for all participants age 15 to 17 who participate in the study. The assent process will follow the same process as the consent process for older youth. The study staff will read the informed consent/assent forms aloud with the potential participant and ask questions about risks and benefits to ensure comprehension.

g. If children are included in the study, how you will obtain permission for them to participate from their parent, legal guardian, or other legal authority (if child is in foster care or under government supervision)

   After determining interest, the study staff member will work with 15 to 17-year-old adolescents to identify the caregiver who will provide consent for their participation. Two different approaches to contact the adult for consent may be employed. One, the parental/caregiver consent form is sent home with the young person to obtain the signature of the adult. This is a common practice within the ART clinics as they send permission slips home for signatures for youth to participate in health facility sponsored youth activities with ALHIV. The parent/caregiver adult will be asked to call the study staff with any questions about the study. Another method that may be used, with the adolescent's permission, is to have the health clinic staff or the study team staff call the adult to explain the study and request written permission. The study team has had success recruiting adolescents and their caregivers using these methods in previous research among this population.

h. If you are seeking a waiver of informed consent or assent, the justification for this request:

   N/A

i. Whether you will include a witness to the consent process and why:
   Witnesses will be included in the consent process in cases where a potential participant is illiterate and unable to read the consent form for his/her self. In such cases a witness, someone at the clinic not involved directly in the study, will be asked to watch the consent process and to sign the consent document to indicate that informed consent was conducted.

j. If the language is unwritten, explain how you will communicate accurate information to potential participants and whether you will use props or audio materials:
   N/A

2. Identify the countries where the research will take place, and the languages that will be used for the consent process.

Country	Consent Document(s) (Adult Consent, Parental Permission, Youth Assent, etc.)	Languages
Zambia	YES_AIM1_2_Assent15-17_2017-09-07_English_TC	English
Zambia	YES_AIM1_2_Assent15-17_2017-09-07_English_Clean	English
Zambia	YES_AIM1_2_Assent15-17_2017-09-07_Bemba	Bemba
Zambia	YES_Aim1_CaregiverPermission_2017-09-07_English_TC	English
Zambia	YES_Aim1_CaregiverPermission_2017-09-07_English_Clean	English
Zambia	YES_Aim1_CaregiverPermission_2017-09-07_Bemba	Bemba
Zambia	YES_AIM1_2_Consent_18-24_2017-09-07_English_TC	English
Zambia	YES_AIM1_2_Consent_18-24_2017-09-07_English_Clean	English
Zambia	YES_AIM1_2_Consent_18-24_2017-09-07_Bemba	Bemba
Zambia	YES_AIM1_CaregiverParticipation_2017-09-07_English	English
Zambia	YES_AIM1_CaregiverParticipation_2017-09-07_Bemba	Bemba
Zambia	YES_AIM2_CaregiverConsent_2017-09-07_English	English
Zambia	YES_AIM2_CaregiverConsent_2017-09-07_Bemba	Bemba
Zambia	YES_AIM2_HCP&YPM&StaffConsent_2018-09-07_English	English
Zambia	YES_AIM1_2_PretestConsent_2017-09_07_English	English
Zambia	YES_AIM1_2_PretestConsent_2017-09_07_Bemba	Bemba

C. **Study Implementation**:

1. Describe the procedures that participants will undergo. If complex, insert a table below to help the reviewer navigate.

Figure 1.

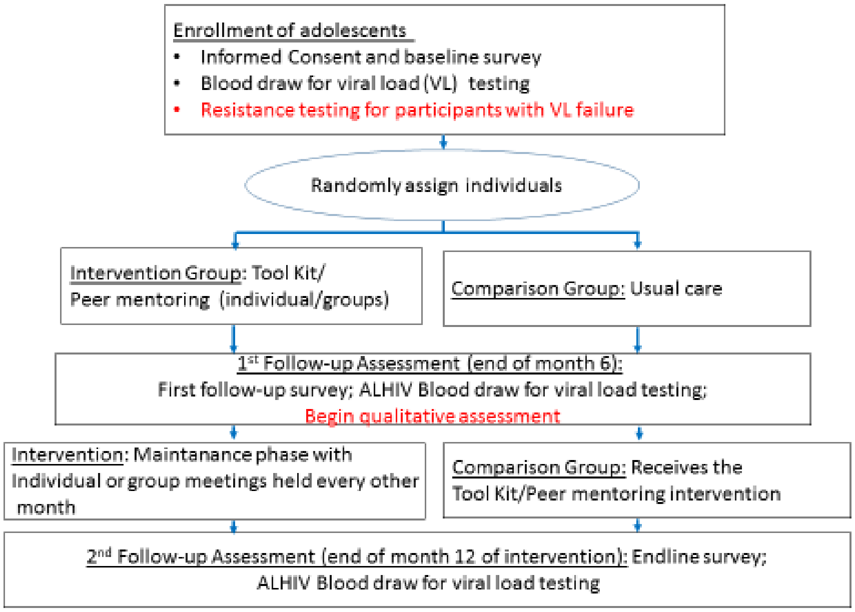

AIM 1: Pilot & Quantitative Evaluation

AIM 1: Assess the impact of peer-mentoring approach to implementing the AIDSTAR-One Toolkit for Transition on viral suppression among ALHIV across different care facility settings (e.g. pediatric hospital, adult hospital, and primary care facilities).

Baseline Data Collection
Once participants are consented and enrolled, they will complete the baseline survey and have their first blood drawn for viral load and, if necessary, resistance testing. To accommodate all literacy levels, study staff will collect survey data in face-to-face interviews with participants. Data collectors will meet with each participant in a private space in or near the health facility to go through survey questions. Surveys will be pre-tested among young adults (18 years and older) to ensure comprehension and acceptability. The baseline survey will collect socio-demographic information and assess a variety of variables that may influence the primary outcome, including internalized stigma, family dynamics, experience learning one's HIV status, alcohol use, mental health, sexual experiences, health communication self-efficacy, HIV disclosure self-efficacy, beliefs about the future, cART adherence, experiences of violence, mindfulness, and HIV treatment self-efficacy.

Random Assignment
After baseline data collection, participants will be randomized to study arms. Participants randomly assigned to the usual care comparison group will receive the regular care offered at their clinic during the first intervention period. For youth who are considered clinically stable, usual care consists of three-month cART prescriptions and quarterly clinic visits. All study sites also offer monthly youth support groups, and periodically offer other activities, such as sports games and group trips, for positive youth within Ndola. Participants randomly assigned to the intervention group will begin the intervention.

Intervention
AIM 1 is to assess the impact of a peer-mentoring approach to implementing the AIDSTAR-One Toolkit for Transition on viral suppression among ALHIV across different care facility settings (e.g. pediatric hospital, adult hospital, and primary care facilities). Peer mentors will be HIV-positive young adults who have successfully transitioned to self-management. These peer mentors will undergo an estimated two-week training by a Training and Capacity Building Specialist to prepare them to be skilled, valued, and paid members of the health care system. As part of their continued training, youth peer mentors will hold practice meetings with youth 18 and older. These youths will be drawn mainly from patients at the clinic whom the research team, including health care providers, know well and who are ineligible for the Project YES! study. For example, this may include participants from a previous study who are ineligible to participate in this research (see exclusion criteria) and who have stayed in contact with the research team. These practice sessions will allow the Youth Peer Mentors to further develop their skills prior to the start of the actual intervention. No data will be collected during these program practice meetings, and the information gained will inform the program only. During program activities, Youth Peer Mentors will utilize toolkit resources to facilitate discussions with youth participants about relevant issues in their lives related to their health.

At the start of the intervention, an introductory meeting will occur with the youth participant, the peer mentor, a health care provider and, if available, an interested caregiver. In the second half of this meeting, the peer mentor will meet with the youth separately to introduce him or her to the Toolkit, which includes a list of tasks adolescents should be comfortable completing as part of successful HIV self-management. We will develop a brief screening tool based on the questions included in the Toolkit (see AIDSTAR-One Toolkit attached) for the peer mentors to use to evaluate the youth's circumstances, indicate what issues may be affecting the youths' adherence, and suggest topics that may be most relevant to discuss in future meetings. The mentor will also use these tools, among others, to develop a personalized action plan with the youth. At this same time, the youth's caregiver (if

present) will meet with the healthcare provider to discuss and answer any questions the caregiver may have about the youth's HIV self-management.

After this introductory meeting, the peer mentor and youth participant will meet every month for one-on-one meetings in or near the health facility for the duration of the six-month intervention. In these one-on-one meetings, the peer mentor will use the toolkit to guide and monitor the youth's movement along the transition continuum and assess the youth's ability to self-manage his/her HIV. Each participant may interact with the toolkit materials in unique ways -- it is intended to be used a resource, rather than a set curriculum. Peer mentors may recommend additional visits with the youth participant, as needed.

To augment this approach, youth participants will also have access to monthly youth group meetings, facilitated by peer mentors, as well as have the option to invite a caregiver to up to three caregiver group meetings over the six-month intervention period.

There will also be specific intervention components for those youth (between the ages of 15 to 24 years) preparing to move from the pediatric/adolescent clinic at ADCH to the adult HIV clinic at NTH. Once a participant is assessed by clinic staff to be ready to transition to the adult setting, they will undergo two preparatory visits at the adult clinic. First, they will visit NTH to tour the facility and meet NTH YPMs and clinic . Second, newly transitioned youths will also have the option to have a YPM or other familiar health staff member attend their first two visits to NTH with them to ease the transition.

Throughout the intervention, we will use several process indicators to track program implementation and participant engagement with intervention components. We will collect attendance information at every one-on-one peer mentoring session, adolescent group session, and caregiver group session. Attendance records from all sessions will be used to examine the relationship between session participation and key outcomes. Peer mentors will document the topics discussed and modules utilized at each one-on-one and group session. This information will inform final analyses and may highlight areas of particular need in this population. We will also document referrals made for additional clinical services, such as mental health services or family planning. This information will provide insight into the additional needs of our study population as well as the ability of program staff to facilitate linkage to appropriate clinical support.

6-month Follow-up Data Collection
After the completion of the first intervention period, all participants will come to the health facility to complete the 6-month follow-up survey and a second blood draw for viral load testing. The 6-month follow-up survey will assess many of the same concepts as the baseline survey in addition to questions about acceptability of the intervention (for intervention arm).

Second Intervention Period (Comparison arm) & Maintenance Period (Intervention arm)
At the end of the primary intervention period and after the 6-month follow-up data has been collected, the primary intervention group will enter a maintenance phase and the comparison group will receive the intervention. This maintenance phase will consist of individual meetings and group meetings every other month. Youth at ADCH who at this point are assessed as ready to transition physically to the adult care clinic will do so and have their maintenance phase at NTH the adult clinic.

12-month Follow-up Data Collection
After the completion of the second intervention period, all participants will come to the health facility to complete the 12-month follow-up survey and a third blood draw for viral load testing. The 12-month follow-up survey will assess many the same concepts as the baseline survey in addition to questions about acceptability of the intervention (for comparison arm).

Medical Chart Review

As part of the informed consent process, all participants will be requested to give permission for study staff to have access to their medical and pharmacy chart information at the HIV clinic, including date of HIV diagnosis, treatment regimen, WHO stages, clinical symptoms, and duration of treatment. Patient medical file data will be collected and entered into a password protected computer in an excel file. Identifying information, such as study ID and patient ID, will be used to link the data collected to merge it with the Magpi data (all this data is entered into secure servers and password protected). Trained study staff will work with the data and access to the secure server is limited to trained research staff. Once the data is merged any identifiers, e.g. patient ID number, will be destroyed to minimize risks to confidentiality.

AIM 2: Qualitative Evaluation
AIM 2: Examine the experiences of adolescents, caregivers, health care providers, peer mentors, and study staff with the peer-mentoring/toolkit intervention.

Youth participants, who provided consent for the qualitative interview when they enrolled, will be reminded of key ethical issues prior to the in-depth interview (e.g. risks, benefits and the right to end the interview when they wish). The caregivers, peer mentors, health care providers, and study staff will also undergo an informed consent process. Trained interviewers will conduct the interviews in a private space in or near the health facility. During the interview, the participants will be asked basic socio-demographic characteristics, including sex, age, HIV status, and relationship to the ALHIV (for caregivers). Participants will also be asked open-ended questions about their experience with the intervention. Youth will additionally be asked about their personal transition to HIV self-management and/or an adult care setting as well as the role of violence victimization in this transition process.

2. Describe the number and type of study visits and/or contacts between the study team and the participant, how long they will last, and where/how they will take place.

AIM 1:
Study visits will take place at or near the health facilities. Intervention arm and Comparison arm participants are required to come to the health facility 11 times (1 introduction meeting, 6 one-on-one meetings, 1 youth group meeting, & 3 data collection visits). However, youth participants in the Intervention group have the opportunity to attend up to 19 scheduled activities during the six-month intervention period and six-month maintenance period (1 introduction meeting, 6 one-on-one meetings, 6 youth group meetings + 3 data collection visits + 3 maintenance meetings). Youth participants in the Comparison group have the opportunity to attend up to 16 scheduled activities (1 introduction meeting, 6 one-on-one meetings, 6 youth group meetings + 3 data collection visits). Youth participants at ADCH who are eligible for transition to NTH will have an additional study visit to tour the facilities at NTH and meet the NTH staff (bringing their total potential visits to 20 for Intervention Arm participants and 17 for Comparison Arm participants). This tour is expected to last about 1 hour.

Peer mentoring one-on-one meetings are anticipated to last between 30 and 90 minutes. Group meetings are anticipated to last between 1 and 2 hours. The baseline data collection is anticipated to last 90 minutes, with the subsequent 6-month and 12- month follow up survey being shorter (fewer questions and randomization will have been completed). Caregivers are not required to participate in any study activities. However, caregivers have the opportunity to participate in up to four scheduled activities (1 introduction meeting & 3 caregiver group meetings).

AIM 2:
One-time in-depth interviews will take place at or near one of the health clinics in a private space and will take about 1.5 hours.

Contacting Participants

Study staff will contact all participants who do not attend a planned study or intervention visit. As part of routine care the clinic staff conducts home visits for YLHIV who have missed appointments to re-engage them in care. The research study will follow similar procedures if a participant misses a study visit. First, attempts will be made to contact the missing participant through the phone numbers and contacts provided during enrollment. During study enrollment, the research team will confirm the appropriate phone numbers and phone ownership (in an earlier study among this population, 69% of 309 youth use cell phones). The team will also request permission to contact the youth's caregiver, or the caregiver's contact, to inquire about the missing participant as well. If the missing participant cannot be reached the trained study interviewers will go to the home to inquire. At the home, the study staff will inquire about the youth using a script, but will not discuss the research study or disclose the youth's participation. If no one is home or they are asked to follow-up later, they will attempt to reach the participant three times before considering the participant lost to follow-up. This process has received approval from the Zambia and Hopkins IRBs for previous studies and has worked well. Participants lost to follow-up will not be replaced in the study.

3. Describe the expected duration of the study from the perspective of the individual participant and duration overall.

   The official study period is from May 15, 2016 to June 30, 2019. From a participant's perspective, the overall duration will be about 18 months – 2 months for recruitment and baseline data collection, 6 months for the initial intervention, 2 months for midline data collection, 6 months for the comparison group to receive the intervention and the initial intervention group to receive maintenance care and 2 months for endline data collection. However, recruitment and follow-up interviews between sessions may take less time.

4. Provide a brief data analysis plan and a description of variables to be derived.

   Aim 1 & Sub-Aim 1A Quantitative Analyses
   The primary analysis to assess impact will use generalized estimating equation (GEE) (22) to compare the intervention to the comparison arm on VL suppression. This process will also be used to examine other key outcomes over time, such as self-reported medication adherence and internalized stigma.

   This approach accounts for within subject correlations due to repeated measures. We will use GEE models with appropriate link functions (e.g., identity for continuous data, logit for binary data) and variance functions (e.g., identity for continuous data, logit for binary data) and variance functions (e.g., normal for continuous data, binomial for binary data). Unstructured working correlation matrices will be used in all models. The primary outcome will be cART adherence as measured by viral load suppression </= 1,000 copies/mL. In the stratified analysis (aims 1A), the models described above will be fit separately to two sub-samples of study participants – those participants who started the intervention in a pediatric care setting (i.e. ADCH) and those participants who started the intervention in an adult care setting (i.e. NTH, Twapia Clinic, and Lubuto Clinic). We will also examine pre- and post-intervention effects, and if outcomes change over time, at the 6-month and 12-month follow-ups. Secondary outcomes will include adolescents' transition status, including how many adolescents physically transitioned to adult care from the children's hospital.

   Descriptive statistical methods will be used to assess the feasibility and acceptability of the intervention. The study team will also analyze attendance and the reasons for missed sessions. Together these data will provide valuable information on the feasibility of the intervention and participant exposure to intervention elements.

   Finally, secondary data analyses will be carried out on participants' reported experiences of violence victimization. Descriptive statistics will be used to obtain weighted estimates of the youth's

lifetime and past-year prevalence of physical, psychological, and sexual violence victimization, with percentages and 95% confidence intervals. Logistic regression will be conducted to examine associations between youth's violence victimization and VL suppression, with GEE models to account for the potential correlations between youth's responses from the same clinic. Associations between violence victimization and VL suppression will further be assessed using propensity scores. Such analyses will shed light on the prevalence of violence victimization and its relationship with VL within a population of youth living with HIV in Zambia, which will inform the potential integration of violence-focused content into the intervention.

Aim 2 Qualitative Analysis
A content analysis approach will be utilized for the qualitative analysis. To facilitate this process, all IDIs will be audio-recorded with participant permission, transcribed, translated, and coded with a qualitative software package, such as Nvivo. These codes will help identify common themes and concepts across interviews (e.g., favorite/least favorite elements of the peer-mentoring intervention). The study team will develop initial codes (e.g., positive perspectives, negative perspectives) but will also work together to identify and build upon themes identified as salient in the text.

5. Answer the following **if they are relevant to your study design**:

A. If the study has different arms, explain the process for assigning participants (intervention/control, case/control), including the sequence and timing of the assignment.

Randomization description
After baseline data collection, study participants will be randomized in 1:1 allocation ratio to receive either peer-mentoring intervention or usual care. Stratified randomly permuted block randomization (block sizes 4 and 6) will be used to generate the randomization scheme. Blocking will facilitate balanced allocation between the intervention arms, and random block size will reduce predictability of the group assignment. Randomization will be stratified by site and by gender, within site, for a total of 16 strata. Random allocation list will be generated, separately for each stratum, using `ralloc' command (23) in STATA statistical software (24). The total sample size is 288 adolescents, but 380 random allocations are created (on average 38 per stratum) to account for potential allocation mistakes or discontinuations. If males are difficult to recruit more random allocations may be generated for females to make sure to reach the total sample size needed.

Allocation Concealment
Random allocation will be concealed from the study team by the use of opaque envelopes that contain the stratum designation (e.g. male, 15-24, ADCH), a sequential number, and, on the inside, in addition to this information, the intervention arm assignment. These envelopes will be stored at the study team offices or the clinic and remain sealed until prospective participants are ready to be randomized. Envelopes are not to be opened until a prospective participant is ready to be enrolled and must be used in numeric order.

After the study staff has confirmed that the prospective participant has met all inclusion criteria and none of the exclusion criteria, informed consent has been obtained, and baseline data collection has occurred, the study staff will open the next envelope and assign treatment allocation to the youth participant. The participant's ID number will then be written on the opened envelope. The opened allocation envelope will be retained for documentation in a secure location out of reach of personnel who should remain blind to participants' assignments.

Blinding

Since the study participants cannot be blinded to the intervention given that they will be attending peer mentor sessions, primary outcome assessors and data analysts will be blinded. Clinical staff who participate in blood draws and viral load testing will not know participants' study group status. Additionally, study groups will be named "A" and "B" instead of "Intervention" and "Comparison" in the database to avoid bias during the primary analysis.

B. If human biospecimens (blood, urine, saliva, etc.) will be collected, provide details about who will collect the specimen, the volume (ml) and frequency of collection, how the specimen will be used, stored, identified, and disposed of when the study is over. If specimens will be collected for use in future research (beyond this study), complete the "Biospecimen Repository" section below.

All participants will have blood drawn at three points in the study – baseline for VL and if needed resistance testing, and 6-month and 12-month follow-up for VL testing only. Baseline samples with viral loads of 1000 copies/ml or higher will undergo resistance testing. Trained professionals will collect about 6ml of blood for each viral load and resistance test (a total 12 ml at baseline, 6 ml for the 6- and 12-month follow-up visits each). Blood samples for viral load testing (and resistance testing when applicable) will be collected at all sites, labeled with an ID number, and securely stored at ADCH.

Viral load testing will use the Roche CAP/CTM HIV-1 Test v2.0. The Arthur Davison Children's Hospital lab is a CDC GAP Proficiency monitored lab that routinely performs VL testing in Zambia as part of clinical care.

Laboratory source data will be retained by the laboratory according to local requirements. We will follow local bio-hazardous materials regulations to ensure blood samples are stored in an appropriate repository and are destroyed after the viral load and resistance tests are completed and verified. Resistance testing will be conducted at a qualified lab, such as ADCH, Lancet, University Teaching Hospital (UTH), Center for Infectious Disease Research in Zambia (CIDRZ).

Clinical results (i.e. viral load and resistance test results) will be maintained in participant files at the clinic with participant consent.

C. If genetic/genomic analyses are planned, address whether the data will be contributed to a GWAS or other large dataset. Address returning unanticipated incidental genetic findings to study participants.

   N/A

D. If clinical or laboratory work will be performed at JHU/JHH, provide the JH Biosafety Registration Number.

   N/A

E. If you will perform investigational or standard diagnostic laboratory tests using human samples or data, clarify whether the tests are validated and/or the lab is certified (for example is CLIA certified in the U.S.). Explain the failure rate and under what circumstances you will repeat a test. For all human testing (biomedical, psychological, educational, etc.), clarify your plans for reporting test results to participants and/or to their families or clinicians. Address returning unanticipated incidental findings to study participants.

Blood samples for viral load resistance testing will be collected at all sites and securely stored at ADCH, labeled with an ID number only. Viral load testing will use the Roche CAP/CTM HIV-1 Test v2.0. The Arthur Davison Children's Hospital lab is a CDC GAP Proficiency monitored lab that routinely performs VL testing in Zambia as part of clinical care. ADCH is also an approved lab for studies like NIH PopART. As such, viral load testing will be performed according to validated SOPs and appropriate quality control measures as practiced by ADCH. In the ADCH lab, the technicians are fully trained and competent in performing VL testing. Laboratory source data will be retained by the laboratory according to local requirements. We will follow local bio-hazardous materials regulations to ensure blood samples are stored in an appropriate repository and are destroyed after the viral load and resistance tests are completed and verified. Resistance testing will be conducted at a qualified lab, such as ADCH, Lancet, University Teaching Hospital (UTH), Center for Infectious Disease Research in Zambia (CIDRZ).

Communicating test results

Test results from each data collection point (baseline, midline and endline) will be placed in participants' medical files for use by their health care providers. In the introductory meeting of the peer mentoring intervention, participants will review their test results with their health care provider and peer mentor (and interested caregiver, if present). Participants in the maintenance phase will also have opportunities to review their midline VL status with the clinic staff and peer mentor. While all results will be placed in participants' medical files, the study team will make sure that health staff are notified as soon as possible of any participant with viral failure who tests positive for resistance whether they are in the comparison or the intervention arm, so that their ART may be adjusted accordingly.

F. If your study involves medical, pharmaceutical or other therapeutic intervention, provide the following information: N/A

a. Will the study staff be blind to participant intervention status?

b. Will participants receive standard care or have current therapy stopped?

c. Will you use a placebo or non-treatment group, and is that justifiable?

d. Explain when you may remove a participant from the study.

e. What happens to participants on study intervention when the study ends?

f. Describe the process for referring participants to care outside the study, if needed.

VI. <u>Data Security and Confidentiality Protections:</u>

A. <u>Personally Identifiable Information (PII)</u>:

Please identify the Personally Identifiable Information (PII) that you may be collecting and using at any of the following stages of your study: ***Recruitment***, ***Consent,*** *and* ***Study Implementation***.

Name, signature, initials, or other identifiable code	☒
Geographic identifier: address, GPS location, etc.	☒
Dates: birth, death, clinical service, discharge, etc.	☒
Contact information: phone numbers, email address, etc.	☒
ID: Social Security Number, driver's license number, etc.	☐
Health record identifiers: medical record, insurance plan number, etc.	☒

Account numbers	☐
Device identifiers: e.g., implants	☐
Internet identifiers: IP address, social media accounts	☐
Biometric identifiers, including finger and voice prints	☐
Audio recordings	☒
Video or full face photographic images	☐
Genomic/genetic data	☐
Any other unique identifying number, characteristic, or code (note: this does not mean the unique code assigned by the investigator to code the data)	☐
Other: Click here to enter text.	☐

B. <u>Recruitment:</u>

Will you collect identifiers for the purpose of contacting potential participants? Yes ☒ No ☐

If **yes**, will you retain the identifiers after the recruitment contact has been made? Yes ☒ No ☐

C. <u>Data Collection:</u>

In what form will you collect and store PII? When you respond, think of PII collected for recruitment, consent, and other study purposes.

1. **<u>Hard Copy/Paper</u>:** Yes ☒ No ☐

   If yes, please answer the following:

   a. How will the data be kept secure during transfer from study collection site to storage site?

      Baseline, six month and endline survey data and qualitative data collection forms and materials, will be taken directly to the study offices located at ADCH. At ADCH they will be kept under lock and key. Youth Peer Mentors will also keep a study binder/folder for each participant to track process indicators (e.g. sessions attended, topics covered, referrals made). These participant folders will be kept in a locked place in the study clinics with access limited to study staff.

   b. Will the data be secured in a locked cabinet or room? Yes ☒ No ☐
   c. Are the data collection forms and study data stored without personal identifiers and separate from the study IDs/code? Yes ☒ No ☐
   d. How long after study completion will you keep the hard copy/paper forms? Identifiable data will be retained until the study is complete and closed.

2. **<u>Electronic</u>:** Yes ☒ No ☐

   If yes, please answer the following:

   a. Will the data be collected/stored on a portable device (laptop, mobile phone, tablet, PDA) protected by encryption? Yes ☒ No ☐

   b. Will the data be stored on a secure server or in the Cloud/Web?
      Secure Server ☒ Cloud/ Web ☐
      Magpi data is uploaded to a secure server. Back up of data will be stored on JHBox.

   c. Will it be encrypted? Yes ☒ No ☐

d. Will you be backing up your data? Yes ☒ No ☐

3. **Audio Recording**: Yes ☒ No ☐

If yes, please answer the following:

a. Will you store the audio recording securely in a locked cabinet/room until transcription is complete?
Yes ☒ No ☐
b. Will the audio recording be destroyed after transcription? Yes ☒ No ☐

If no, why not?

4. **Photograph/Video**: Yes ☐ No ☒

If yes, please answer the following:

a. Will the photographs/videos be stored securely in a locked cabinet or room? Yes ☐ No ☐

b. Will the photograph/video be destroyed? Yes ☐ No ☐

If yes, when?

D. PII De-Identification of Data Used for this Study:

When will you destroy the PII and/or the code linking the PII with the study ID?
The codes will be destroyed after data cleaning and analysis is complete.

E. Data Storage and Analysis:

One of the keys to protecting PII is the proper use of tools to share and conduct your analysis. JH and JHSPH offers several options for you to consider. Please select the system that you plan to use to protect your study data by clicking the box. Consult JHSPH IT for assistance if needed.

☐ **JH Virtual Desktop**: IT@JH provides (for a monthly fee) a virtual Windows desktop.
☐ **JHSPH SharePoint and File Shares**: These systems provide a managed and secure platform for your research project. They also provide a built-in encrypted backup solution.
☐ **JHSPH RedCAP or HPCC**: These are departmentally managed applications.
☒ **JHBox**: Johns Hopkins Box (JHBox) is a secure cloud-based file sharing and file storage service.
☐ **Independent Departmental Servers and Systems**: These servers are typically managed by departmental or research team IT staff.
☒ **Other**: Please provide details regarding any other systems being utilized. Magpi

Johns Hopkins Global mHealth Initiative, of which Dr. Denison is a member, has an enterprise license to Magpi, a widely used mobile data collection system. The system allows data collectors to use customized digital forms to record information that is subjected to error checks (range, validation, etc.) in real-time at the point of data collection, substantially reducing the risk of data entry errors and dramatically shortening the time to data analysis. We anticipate using Magpi for data collection. Study staff will be trained on data collection using Magpi and paper form. Dr. Denison has successfully used Magpi as part of her CFAR faculty development award and other research in Zambia. All mobile devices will be password protected and Magpi data is automatically uploaded to a secure server. Magpi is also compatible with a HIPAA-compliant system.

F. <u>Other Data Security Measures:</u>
In addition to the details regarding data collection, please review the following questions. This additional information will be utilized to assist in the development of a comprehensive Data Security plan. This would include the systems used to analyze the data, data security contacts and additional requirements.

1. Do you have a designated person on your research team other than the PI who is the technical contact for a Data Security plan? Yes ☐ No ☒
   If yes, please provide a contact name:

2. Does your sponsor have other specific data security requirements for the study data? Yes ☐ No ☒
   If possible, please explain:

3. Please add any other information that you believe is relevant to data security.

G. <u>Certificate of Confidentiality</u>:

Will the study data stored in the **United States** be protected by a Certificate of Confidentiality?

If yes, explain who will apply for and maintain the Certificate.
(http://grants.nih.gov/grants/policy/coc/appl_extramural.htm)

H. Will you use clinical data of 500 records or more from Johns Hopkins Hospital and its affiliates?
Yes ☐ No ☒

If yes, please complete the JHM Data Security Checklist available on the JHSPH IRB website: www.jhsph.edu/irb and upload a copy of the checklist to the "Miscellaneous" section.

VII. <u>Risks of the Study</u>:

A. Describe the risks, discomforts, and inconveniences associated with the study and its procedures, including physical, psychological, emotional, social, legal, or economic risks, and the risk of a breach of confidentiality. These risks should be described in the consent documents.

A potential risk of being involved in the intervention pilot or the in-depth interviews is other people finding out about the youth's HIV status. Due to the group nature of the peer and caregiver support groups, the youth's HIV status will be known to others in the group. Unwanted disclosure could lead to stigma and discrimination including verbal and physical abuse.

Additional risks include subjects feeling embarrassed or uncomfortable by some of the questions on the enrollment and follow-up surveys. Adolescents who take part in viral load testing may also experience some discomfort during the blood draws. Very rarely people may faint or get an infection at the blood draw site.

B. Describe the anticipated frequency and severity of the harms associated with the risks identified above; for example, if you are performing "x" test/assessment, or dispensing "y" drug, how often do you expect an "anticipated" adverse reaction to occur in a study participant, and how severe do you expect that reaction to be?

While the severity of unintended disclosure and/or breach of confidentiality is potentially great, the anticipated frequency of these events is low, both based on the published literature around peer groups for ALHIV, as well as based on the study clinic's experiences providing support groups for ALHIV and tracing youth in their homes and our prior intervention-based research with this population

The anticipated severity of the harms associated with a blood draw is low and would only happen when blood is drawn at three points in the study.

C. Describe steps to be taken to minimize risks. Include a description of your efforts to arrange for care or referral for participants who may need it.

All study staff will be trained on research ethics and will be trained on the importance of informed consent and confidentiality. Hard copies of data will be stored in locked cabinets and all electronic data will be stored on password-protected computers only accessible to study staff. To ensure confidentiality, the Magpi electronic devices will be password-protected. After the data have been downloaded, they will be sent to the study PI and study coordinator at JHSPH through a secure server, such as JHBox. The study team will run data verification checks to ensure that the submitted data is complete and valid. Access to all study databases will be password protected to ensure the confidentiality of participants. Data collection instruments, utilization logs, consent forms, adverse event reports and equipment will be stored in locked cabinets/containers in the study clinics or the main study office

Blood draws will be conducted by trained professionals. In the case of an adverse reaction during a blood draw, participants or study staff can contact Dr. Mwansa directly and he will ensure the participant receives necessary care. Referral systems are also in place for any mental health support needs.

Referrals

If during the study, study staff members (data collectors, administrators, or peer mentors) have concerns about a youth participant's wellbeing, they will refer the youth to clinical staff (such as the sister-in-charge) who will then be able to connect the youth with appropriate care. Study staff will also automatically refer participants to a clinic staff member if any of the following is reported during data collection: a) any experience of severe violence in the past year b) any experience of sexual violence ever or c) thoughts of suicide in the past week. Participants 18 years and older have the option to refuse to talk with a healthcare provider. Cases of severe physical or sexual violence occurring in the past month or thoughts of suicide in the past week will be prioritized, should treatment (e.g. post-exposure prophylaxis for HIV) and/or immediate counseling be needed. Clinic staff will respond according to clinical practice, local policy and Zambian law. ADCH and NTH provide mental health services and ADCH has a trained clinician available to respond to sexual violence cases at all times.

The study staff member who makes the initial referral to clinic staff will complete a Referral Form that tracks the reason for referral and name of the clinical staff member contacted. Study staff will also do their best to track the outcome of these referrals to ensure that participants receive appropriate care. To minimize the risk of unintended disclosure or breach of confidentiality we will only record the participant's initials on the Referral Form. In cases where participants have the same initials, we will collect the first two letters of the first name as well as the first initial of the last name to avoid confusion. Referral forms will be stored in participant folders in a locked place at the clinics.

As part of regular clinic procedures and to support the tracking of referrals, each clinic will be given a tracking notebook to record the participant's name, date of referral, priority of referral, and action taken. This tracking notebook will be kept in a locked cabinet or room with access limited to the study staff

This referral process has been developed in partnership with Dr. Jonathan Mwansa, the co-PI and Dr. Sam Miti, a co-investigator on this study. Both are experts in working with violence- and HIV-positive youth, having previously run the One Stop Child Sexual Abuse Center in Lusaka.

D. Describe the research burden for participants, including time, inconvenience, out of pocket costs, etc.

All study visits will be held at or near the clinic/study site from which the participant is recruited (or at the clinic to which the youth has transitioned, as will be the case for some youth recruited at ADCH but transitioned to NTH)Enrolled participants are required to attend an introductory meeting (1), six monthly one-on-one meetings with peer mentors (6), the first youth support group meeting (1), and three data collection events (3) (Total of 11 required study visits). Participants at ADCH who transition to NTH will also attend a tour of NTH. All other activities, including additional group meetings and maintenance meetings, are optional.

E. Describe how participant privacy will be protected during data collection if sensitive questions are included in interviews.

Only trained study staff will be interacting with study participants and data, with an emphasis on hiring staff with experience working with youths. All interviews will be conducted in a private location at or near the study health clinics with trained interviewers. All the information collected is for research purposes only and data will be kept in the strictest confidence.

Contacting Caregivers
Contact with a caregiver will only be made with the explicit consent of the adolescent. Study team staff will work with youth participants to identify the caregiver who will be invited to participate in caregiver study activities (i.e. introductory meeting, caregiver group meetings, in-depth interview) and the best way of contacting that adult. In previous research the caregiver contacted was often the same person listed at the clinic as a treatment supporter. Treatment supporters are the adult contact on file at the clinic and the person who actively supports the young person's care and treatment. The clinic does contact treatment supporters as needed when working with an adolescent. The study team has successfully recruited and enrolled adolescents and their caregivers in Ndola, Zambia using these procedures and will use previously trained study personnel when possible.

VIII. <u>Direct Personal and Social Benefits</u>:

A. Describe any potential direct benefits the study offers to participants ("payment" for participation is not a direct personal benefit).

There are no direct benefits for participants. Adolescent subjects in the study will receive three viral load tests, and if found to have a viral load equal to or exceeding 1000 copies/ml at baseline, a resistance test. This information will be shared with their health care providers to inform the youth's ongoing treatment. Participants may enjoy talking with others in one-on-one and group study activities about their experiences. Participants may benefit in the future from improved services based on the knowledge gained from this study.

B. Describe potential societal benefits likely to derive from the research, including value of knowledge learned.

This will be one of the first operational research study to refine and test strategies for improving youths' transition into adult HIV self-management in SSA, even though around 80% of the world's HIV-positive adolescents reside in SSA (25). Results from this study will offer badly needed evidence-based guidance and refined tools for YLHIV, their families, and their health care providers to improve the care transition process and related HIV outcomes. Specific materials produced will include recommendations for effective implementation of the AIDSTAR-One toolkit application, a curriculum for training peer mentors, and data on strategy effectiveness to inform programs and policies for YLHIV in SSA and globally.

IX. <u>Payment</u>:

A. Describe the form, amount, and schedule of payment to participants. Reimbursement for travel or other expenses is not “payment,” and if the study will reimburse, explain.

Participants will not be paid, but they will be reimbursed.

AIM 1: Participants will be reimbursed 50 Kwacha (approximately $5 USD) in cash per scheduled, study-related visit (i.e. introductory meeting, one-on-one meetings, group meetings, data collection events). This payment is reimbursement for the participants’ time and transport costs.
AIM 2: All youth and caregiver participants will similarly be compensated 50 Kwacha in cash for their participation in an in-depth interview for their time and transport costs.

B. Include the possible total remuneration and any consequences for not completing all phases of the research.

AIM 1: If youth participants in the Intervention group or Comparison group attend all required visits and no additional visits (1 introduction meeting, 6 one-on-one meetings, 1 youth group meeting + 3 data collection visits), they can earn up to 550 Kwacha. If participants in the Intervention group attend all possible scheduled activities during both the six-month intervention period and six-month maintenance period (1 introduction meeting, 6 one-on-one meetings, 6 youth group meetings + 3 data collection visits + 3 maintenance one-on-one meetings, 3 youth group meetings) they can earn up to 1050 Kwacha. If youth participants in the Comparison group attend all possible scheduled activities (1 introduction meeting, 6 one-on-one meetings, 6 youth group meetings + 3 data collection visits), they can earn up to 800 Kwacha. Participants who transition to NTH and attend the tour of the facility will be reimbursed an additional 50 Kwacha for their travel.

Caregivers are not required to participate in any study activities. However, if caregivers of a youth in the Intervention group or Comparison group participate in all possible scheduled activities (1 introduction meeting, 3 caregiver group meetings), they can earn up to 200 Kwacha.

AIM 2: If a youth or caregiver participates in an in-depth interview, he/she can earn an additional 50 Kwacha.

All participants are provided reimbursement after the study visits end. For longer study visits, participants may also be provided with a refreshment.

X. Study Management:

A. Oversight Plan:

1. Describe how the study will be managed.

   The two study PIs will oversee the overall implementation of the research project (Dr. Mwansa in Zambia and Dr. Denison based at JHU in Baltimore). Please see Question 4 below for additional info on remote supervision and communication plans.

2. What are the qualifications of study personnel managing the project?

   - Dr. Julie Denison, the PI in the U.S., has a doctoral degree in International Public Health from the Hopkins Bloomberg School of Public Health and over 15 years of research experience with youth in Zambia.
   - Dr. Jonathan Mwansa, the co-PI, and Dr. Sam Miti, a co-investigator on this study, both have extensive experience working with youth living with HIV and are both experts in working with youth who have experienced violence-, having both previously run the One Stop Child Sexual Abuse Center in Lusaka.

- o Virginia Burke, the Senior Research Coordinator in the U.S., has a MSPH degree from Hopkins, experience administering surveys in clinical settings, and qualitative data collection and analysis expertise.
- o Christy Frimpong, the Program Implementation Manager, has an MPH in Epidemiology and Biostatistics from the University of Zambia and over two years of research experience.
- o Christine Jere, the Research Coordinator in Zambia, has a bachelor's degree in development studies from the University of Zambia, a transcript diploma in social work, and experience working with the PI on previous research with youth living with HIV.
- o Lindy Imboela Mbando has a diploma in social work from the University of Zambia and has worked with the PI on previous research with youth living with HIV.
- o Kate Merrill, a doctoral student in the SBI IH program, has an MSc in Epidemiology from the London School of Hygiene and Tropical Medicine, experience administering surveys in clinical settings, and quantitative and qualitative data collection and analysis expertise.

3. How will personnel involved with the data collection and analysis be trained in human subjects research protections? (Use the JHSPH Ethics Field Training Guide available on the JHSPH IRB website: www.jhsph.edu/irb.)

   All study staff will complete an accepted ethics training program such as the Collaborative Institutional Training Initiative (CITI) online human subject's research ethics training program. All study staff will also undergo an in-person study training that will cover ethics and research implementation prior to study implementation.

4. If the PI will not personally be on-site throughout the data collection process, provide details about PI site visits, the supervision over consent and data collection, and the communication plan between the PI and study team.

   We have a management structure in country to monitor and oversee ethical conduct of the study. The co-PI, Dr. Mwansa, and co-Investigator, Dr. Miti, are both based at ADCH and will provide general oversight and management of study operations in country. We also have an Implementation Manager who will monitor human subjects' protections and program implementation and a Research Coordinator and Senior Research Assistant who will supervise and coordinate recruitment and ensure data quality and adherence to study protocol. These staff will determine that all regulatory requirements are met in Zambia.

   While the JHSPH PI will not personally be on site throughout data collection, there are several mechanisms that will ensure oversight. There will be weekly team calls with the JHSPH PI to review weekly data reports and address concerns and challenges as they arise. The Hopkins Senior Research Coordinator will work with in-country staff to monitor the data as they come in to ensure completeness and accuracy. Both the JHSPH PI and Senior Research Coordinator will conduct site visits to train staff and to monitor implementation. During these trips, they will visit the study sites, oversee program implementation, check data management systems for completeness, and ultimately to facilitate research dissemination. Dr. Denison will also be traveling twice to South Africa for another project, which may give her opportunities to add on additional site visits to Ndola.

B. **Recordkeeping:**
Describe how you plan to ensure that the study team follows the protocol and properly records and stores study data collection forms, IRB regulatory correspondence, and other study documentation. For assistance, contact housecall@jhu.edu.

Recordkeeping and study procedures will be monitored several ways. First, the study PI will hold weekly meetings with the study team to review progress and challenges (as well as be available either

via phone or email on all other days during study implementation). Second, the PI and Study Coordinator will perform QA and QC measures for each participant's data submission. This includes checking for missing fields, legibility, staff signatures, and (if applicable) recurring issues. Quality assurance will involve engaging in good data management activities, such as checking the integrity of data storage and examining frequency distributions to look for anomalies such as an excessive number of "don't know" responses or problems with skip patterns will be in place.

C. **Safety Monitoring:**

1. Describe how participant safety will be monitored as the study progresses, by whom, and how often. Will there be a medical monitor on site? If yes, who will serve in that role?

The PIs, as well as the Implementation Manager and Co-investigator in country, will monitor human subjects' protections on an ongoing basis. In addition, they will also review participant referrals and any other safety issues at weekly staff meetings to ensure study procedures are appropriately followed. These weekly meetings will continue for the duration for the study. Any study staff who have concerns about the wellbeing of a youth participant will complete a referral form and connect the youth to a healthcare provider at their clinic.

2. If a Data Safety Monitoring Board (DSMB), or equivalent will be established, describe the following:

   a. The DSMB membership, affiliation and expertise.
      N/A

   b. The charge or charter to the DSMB.
      N/A

   c. Plans for providing DSMB reports to the IRB.
      N/A

3. Describe plans for interim analysis and stopping rules, if any.
   N/A

D. **Reporting Unanticipated Problems/Adverse Events (AE's) to the IRB (*all studies must complete this section*)**:

Describe your plan for reporting to the IRB and (if applicable) to the sponsor. Include your plan for government-mandated reporting of abuse or illegal activity.

Potential protocol violations include a breach in confidentiality. Research staff will be trained on the study protocol, procedures and ethical issues, including the kinds of activities that constitute a protocol violation and the procedure for violations and adverse events. Staff will be requested to report all protocol violations and adverse events to the Program Implementation Manager immediately. The Program Implementation Manager will then immediately inform the study co-PIs, co-investigator, and JHU Study Coordinator. The JHU PI will work with the ADCH co-PI to inform Eres Converge and the Johns Hopkins Bloomberg School of Public Health's Institutional Review Board. Zambia's ethics committee's procedures will be followed for reporting protocol violations in Zambia.

If during the study, study staff members (data collectors, administrators, or peer mentors) have concerns about a youth participant's wellbeing, they will refer the youth to clinical staff (such as the sister-in-charge) who will then be able to connect the youth with appropriate care. The referral process at the clinic will follow clinical practice, local policy and Zambian law (see description under risks detailed above).

NOTE: The IRB does not require submission for all AEs, only those that are **unanticipated, pose risk of harm to participants or others, and are related to the study**.

E. **Other IRBs/Ethics Review Boards:**
If other IRBs will review the research, provide the name and contact information for each IRB/ethics review board and its Federal Wide Assurance, if it has one (available on OHRP's website at http://www.hhs.gov/ohrp/assurances).

Eres Converge
33 Joseph Mwilwa Road
Rhodes Park, Lusaka, Zambia
eresconverge@yahoo.co.uk
+260-955-155633
+260 955 155634

Population Council*
Dr. John Bongaarts
One Dag Hammarskjold Plaza, 3rd Floor, New York, NY 10017
jbongaarts@popcouncil.org
+1 212 339 0660

*Funding for the study is coming through Project SOAR, a Population Council-funded project; Population Council will defer to JHU ethics approval.

F. **Collaborations with non-JHSPH Institutions:**
For studies that involve collaboration with non-JHSPH institutions, complete the chart below by describing the collaboration and the roles and responsibilities of each partner, including the JHSPH investigator. This information helps us determine what IRB oversight is required for each party. Complete the chart for all multi-collaborator studies.

Insert Name of Institutions in Partner column(s); add additional columns if necessary.

	JHSPH	Arthur Davison Children's Hospital
Primary Grant Recipient	Julie Denison (PI)	
Collaborator		Dr. Mwansa (consultancy), Arthur Davison Children's Hospital (co-investigator)

For the following, indicate "P" for "Primary", "S" for "Secondary" (as appropriate to role and level of responsibility.) Add additional items if useful.

1.	Human subjects research ethics training for data collectors	P	P
2.	Day to day management and supervision of data collection	S	P
3.	Reporting unanticipated problems to the JHSPH IRB/Sponsor	P	S
4.	Hiring/supervising people obtaining informed consent and/or collecting data	S	P

5.	Execution of plan for data security/protection of participant data confidentiality, as described in the Data Security and Confidentiality Protections section above.	P	P
6.	Biospecimen processing, storage, management, access, and/or making decisions about future use	S	P

COMPLETE THE FOLLOWING SECTIONS WHEN RELEVANT TO YOUR STUDY:

XI. Secondary Data Analysis of Existing Data:

A. **Study Design**:

1. Describe your study design and methods. The study design must relate to your stated aims/objectives.

2. Provide an estimated sample size and an explanation for that number.

3. Provide a brief data analysis plan and a description of variables to be derived.

B. **Participants:**

1. Describe the subjects who provided the original data and the population from which they were drawn.

2. If you are receiving, accessing, or using data from a U.S. health care provider, the need for HIPAA review is likely. If you plan to bring identifiable health information from a foreign country to a U.S. covered entity (e.g., lab at the Hopkins SOM), HIPAA may be triggered. If either of these conditions is met, check "yes" to the HIPAA question in the PHIRST application.

3. If you plan to analyze human specimens or genetic/genomic data, provide details about the source of those specimens and whether they were collected using an informed consent document. If yes, explain whether your proposed use is "consistent with" the scope of the original consent, if it potentially introduces new analyses beyond the scope of the original consent, and/or if it introduces new sensitive topics (HIV/STDs, mental health, addiction) or cultural/community issues that may be controversial.

4. Explain whether (and how) you plan to return results to the participants either individually or as a group.

XII. Oversight Plan for Student-Initiated Studies:

A. For student-initiated studies, explain how the PI will monitor the student's adherence to the IRB-approved research plan, such as communication frequency and form, training, reporting requirements,

and anticipated time frame for the research. Describe who will have direct oversight of the student for international studies if the PI will not personally be located at the study site, and their qualifications.

B. What is the data custody plan for student-initiated research? *(Note: Students may not take identifiable information with them when they leave the institution.)*

XIII. Creation of a Biospecimen Repository:

Explain the source of the biospecimens, if not described above, what kinds of specimens will be retained over time. Clarify whether the specimens will be obtained specifically for repository purposes, or will be obtained as part of the core study and then retained in a repository.

A. Describe where the biospecimens will be stored and who will be responsible for them.

B. Describe how long the biospecimens will be stored, and what will happen at the end of that period.

C. Explain whether the biospecimens will be shared with other investigators, inside and outside of JHU, how the decision to share will be made, and by whom. Include your plans, if any, for commercial use. Also explain how downstream use of the specimen will be managed, and what will happen to left-over specimens.

D. Describe whether future research using the biospecimens will include specimen derivation and processing (cell lines, DNA/RNA, etc.), genomic analyses, or any other work which could increase risk to participants. Explain what additional protections will be provided to participants.

E. If future research could yield unanticipated incidental findings (e.g., an unexpected finding with potential health importance that is not one of the aims of the study) for a participant, do you intend to disclose those findings to the study participant? Please explain your position.

F. Explain whether the specimens will be identifiable, and if so, how they will be coded, who will have access to the code, and whether the biospecimens will be shared in linked (identifiable) form.

G. Explain whether the repository will have Certificate of Confidentiality protections.

H. Explain whether a participant will be able to withdraw consent to use a biospecimen, and how the repository will handle a consent withdrawal request.

I. Describe data and/or specimen use agreements that will be required of users. Provide a copy of any usage agreement that you plan to execute with investigators who obtain biospecimens from you.

XIV. Data Coordinating Center:

Complete if JHSPH serves as the Data Coordinating Center.

A. How will the study procedures be developed?

B. How will the study documents that require IRB approval at each local site be developed? Will there be some sort of steering or equivalent committee that will provide central review and approval of study documents, or will template consent forms, recruitment materials, data collection forms, etc. be developed by and provided to the local sites by the coordinating center without external review?

C. Will each local clinical site have its own IRB with an FWA? State whether the coordinating center will collect IRB approvals and renewals from the clinical centers; if not, explain why.

D. How will the coordinating center provide each local site with the most recent version of the protocol and other study documents? What will be the process for requesting that these updates be approved by local clinical center IRBs?

E. What is the plan for collecting data, managing the data, and protecting the data at the coordinating center?

F. What is the process for reporting and evaluating protocol events and deviations from the local sites? Who has overall responsibility for overseeing subject safety: the investigators at the recruitment site, the Coordinating Center, the Steering Committee, or a Data and Safety Monitoring Board (DSMB)? Is there a DSMB that will evaluate these reports and provide summaries of safety information to all the reviewing IRBs, including the coordinating center IRB? Please note that if there is a DSMB for the overall study, then the coordinating center PI does not have to report to the coordinating center IRB each individual adverse event/problem event that is submitted by the local site PIs.

G. Some FDA regulated studies have different AE reporting criteria than that required by the IRB (IRB Policy No. 103.06). How will you reconcile the different requirements, and who is responsible for this reconciliation?

H. Who is responsible for compliance with the study protocol and procedures and how will the compliance of the local sites be monitored and reviewed? How will issues with compliance be remedied?

XV. <u>Drug Products, Vitamins, Food and Dietary Supplements:</u>

Complete this section if your study involves a drug, botanical, food, dietary supplement or other product that will be applied, inhaled, ingested or otherwise absorbed by the study participants. If you will be administering drugs, please upload the product information.

A. List the name(s) of the study product(s), and the manufacturer/source of each product.

**Name of Study Product**	**Manufacturer/Source**

B. List each study product by name and indicate its approved/not approved status.

Approved by the FDA and Commercially Available	Approved by Another Gov't Entity (provide name)	Cleared for Use at Local Study Site

C. If your study product has an Investigational New Drug (IND) application through the U.S. Food and Drug Administration, provide the IND number, the Investigators Brochure and complete and upload into PHIRST the Drug Data Sheet available on the JHSPH IRB website www.jhsph.edu/irb.

D. If your study product is a marketed drug, provide the package inserts or other product information. If the study product WILL NOT be used for its approved indication, dose, population, and route of administration, provide a detailed rationale justifying the off label use of the study product.

E. If the study product is not an FDA approved drug, and is being used without an IND (e.g., dietary supplements, botanicals, etc.), provide safety information (as applicable) and a certificate of analysis.

F. Explain who will be responsible for drug management and supply, labeling, dispensing, documentation and recordkeeping.

G. What drug monitoring and/or regulatory oversight will be provided as part of the study?

XVI. Medical Devices:

Complete this section if your study will involve an approved or investigational medical device (diagnostic, non-significant risk, significant risk).

A. List the name(s) of the study product(s), the manufacturer/source of each product, and whether or not it is powered (electric, battery). Provide product information. If it is electric, upload documentation of clinical engineering approval.

Name of Study Product	Manufacturer/Source	Powered?

B. List each study product by name and indicate its status as approved by a government authority or not approved.

Approved by the FDA and Commercially Available	Approved by Another Gov't Entity (provide name and approval information)	Not Approved

C. If your investigational device is Exempt from the FDA IDE regulations, explain which section of the code applies to your device and why it meets the criteria provided. If it is a diagnostic device, provide pre-clinical information about the sensitivity and specificity of the test and the anticipated failure rate. If you plan to provide the results to participants or their physicians, justify doing so, and explain how those results will validated (or not) against the current "gold standard".

D. If you believe the investigational device is not IDE exempt under 21CFR 812.2(c), but is a "Non-Significant Risk" device considered to have an approved IDE application, provide information from the manufacturer supporting that position.

E. If you are using an investigational device that is a Significant Risk Device, provide the IDE number given by the FDA, or if not under FDA jurisdiction, explain why it is appropriate to use this device in this study. Provide a description of the device, and upload a picture or manufacturing schematics into PHIRST. Provide any other information relevant to a determination of its safety to be used for the purposes outlined in this research plan.

REFERENCES:

1. Amzel A, Toska E, Lovich R, Widyono M, Patel T, Foti C, et al. Promoting a combination approach to paediatric HIV psychosocial support. AIDS. 2013;27:S147-S57.
2. Sawyer SM, Drew S, Yeo MS, Britto MT. Adolescents with a chronic condition: challenges living, challenges treating. The Lancet. 2007;369(9571):1481-9.
3. Rosen DS, Blum RW, Britto M, Sawyer SM, Siegel DM. Transition to adult health care for adolescents and young adults with chronic conditions: position paper of the Society for Adolescent Medicine. Journal of Adolescent Health. 2003;33(4):309-11.
4. Hussen SA, Chahroudi A, Boylan A, Camacho-Gonzalez AF, Hackett S, Chakraborty R. Transition of youth living with HIV from pediatric to adult-oriented healthcare: a review of the literature. Future virology. 2014;9(10):921-9.
5. Bal MI, Sattoe JN, Roelofs PD, Bal R, van Staa A, Miedema HS. Exploring effectiveness and effective components of self-management interventions for young people with chronic physical conditions: A systematic review. Patient education and counseling. 2016.
6. Lorig KR, Holman HR. Self-management education: history, definition, outcomes, and mechanisms. Annals of behavioral medicine. 2003;26(1):1-7.
7. Sattoe JN, Bal MI, Roelofs PD, Bal R, Miedema HS, van Staa A. Self-management interventions for young people with chronic conditions: a systematic overview. Patient education and counseling. 2015;98(6):704-15.
8. Hausner D, Cornman H, Duffy M. Toolkit for transition of care and other services for adolescents living with HIV: Kenya pilot evaluation. 2013.
9. Futterman D, Shea J, Besser M, Stafford S, Desmond K, Comulada WS, et al. Mamekhaya: a pilot study combining a cognitive-behavioral intervention and mentor mothers with PMTCT services in South Africa. AIDS care. 2010;22(9):1093-100.
10. Denison JA, Tsui S, Bratt J, Torpey K, Weaver MA, Kabaso M. Do peer educators make a difference? An evaluation of a youth-led HIV prevention model in Zambian Schools. Health education research. 2012;27(2):237-47.
11. Kim MH, Mazenga AC, Yu X, Ahmed S, Paul ME, Kazembe PN, et al. High self-reported non-adherence to antiretroviral therapy amongst adolescents living with HIV in Malawi: barriers and associated factors. Journal of the International AIDS Society. 2017;20(1):21437.

12. Pantelic M, Boyes M, Cluver L, Meinck F. HIV, violence, blame and shame: pathways of risk to internalized HIV stigma among South African adolescents living with HIV. Journal of the international AIDS society. 2017;20(1):21771.
13. Dow DE, Turner EL, Shayo AM, Mmbaga B, Cunningham CK, O'Donnell K. Evaluating mental health difficulties and associated outcomes among HIV-positive adolescents in Tanzania. AIDS care. 2016;28(7):825-33.
14. Woollett N, Cluver L, Bandeira M, Brahmbhatt H. Identifying risks for mental health problems in HIV positive adolescents accessing HIV treatment in Johannesburg. Journal of Child & Adolescent Mental Health. 2017;29(1):11-26.
15. Gari T, Habte D, Markos E. HIV positive status disclosure among women attending art clinic at Hawassa University Referral Hospital, South Ethiopia. East African Journal of Public Health. 2010;7(1).
16. Maeri I, El Ayadi A, Getahun M, Charlebois E, Akatukwasa C, Tumwebaze D, et al. “How can I tell?” Consequences of HIV status disclosure among couples in eastern African communities in the context of an ongoing HIV “test-and-treat” trial. AIDS care. 2016;28(sup3):59-66.
17. Watt MH, Dennis AC, Choi KW, Ciya N, Joska JA, Robertson C, et al. Impact of sexual trauma on HIV care engagement: Perspectives of female patients with trauma histories in Cape Town, South Africa. AIDS and Behavior. 2017;21(11):3209-18.
18. Iliyasu Z, Abubakar IS, Babashani M, Galadanci HS. Domestic violence among women living with HIV/AIDS in Kano, Northern Nigeria. African Journal of Reproductive Health. 2011;15(3):43-53.
19. Hatcher AM, Turan JM, Leslie HH, Kanya LW, Kwena Z, Johnson MO, et al. Predictors of linkage to care following community-based HIV counseling and testing in rural Kenya. AIDS and Behavior. 2012;16(5):1295-307.
20. Lichtenstein B. Domestic violence in barriers to health care for HIV-positive women. AIDS Patient Care & STDs. 2006;20(2):122-32.
21. Hatcher AM, Smout EM, Turan JM, Christofides N, Stöckl H. Intimate partner violence and engagement in HIV care and treatment among women: a systematic review and meta-analysis. Aids. 2015;29(16):2183-94.
22. Diggle P, Liang K, Zeger S. Analysis of longitudinal data oxford statistical science series 13. Clarendon Press, Oxford; 1994.
23. Ryan P. RALLOC: Stata module to design randomized controlled trials. 2011.
24. StataCorp. Stata Statistical Software: Release 14. StataCorp LP; 2015.
25. UNICEF. For Every Child, End AIDS -- Seventh Stocktaking Report. New York, NY: UNICEF; 2016.
